# Short‐chain fatty acids in multiple sclerosis: Associated with disability, number of T2 lesions, and inflammatory profile

**DOI:** 10.1002/acn3.52259

**Published:** 2025-03-03

**Authors:** Maria Inmaculada Dominguez‐Mozo, Daniel López‐Mecández, Luisa María Villar, Lucienne Costa‐Frossard, Noelia Villarrubia, Yolanda Aladro, Belén Pilo, Xavier Montalbán, Manuel Comabella, Ignacio Casanova‐Peño, Inés González‐Suárez, María Luisa Martínez‐Ginés, Jose Manuel García‐Domínguez, Estefanía García‐Calvo, Andrés Machuca‐Marcos, Jose Luis Luque‐Garcia, María Angel Garcia‐Martinez, Rafael Arroyo, Roberto Alvarez‐Lafuente

**Affiliations:** ^1^ Grupo de Investigación de Factores ambientales en enfermedades degenerativas, Instituto de Investigación Sanitaria del Hospital Clínico San Carlos (IdISSC), Red de Enfermedades Inflamatorias (REI), Red Española de Esclerosis Múltiple Madrid Spain; ^2^ Department of Clinical Analysis Hospital Clínico San Carlos, Instituto de Medicina del Laboratorio (IML) Madrid Spain; ^3^ Servicio de Inmunología, Hospital Universitario Ramón y Cajal, Red de Enfermedades Inflamatorias (REI), Red Española de Esclerosis Múltiple Madrid Spain; ^4^ Servicio de Neurología, Hospital Universitario Ramón y Cajal, Red de Enfermedades Inflamatorias (REI), Red Española de Esclerosis Múltiple Madrid Spain; ^5^ Servicio de Neurología, Hospital Universitario de Getafe Spain; ^6^ Servei de Neurologia, Centre d'Esclerosi Múltiple de Catalunya (Cemcat), Institut de Recerca Vall d'Hebron (VHIR), Hospital Universitari Vall d'Hebron, Universitat Autònoma de Barcelona Barcelona Spain; ^7^ Department of Neurology, Torrejón de Ardoz, España, School of Medicine, University Hospital Torrejón Francisco de Vitoria University Madrid Spain; ^8^ Unidad de enfermedades desmielinizantes, Hospital Álvaro Cunqueiro, Red de Enfermedades Inflamatorias (REI) Vigo Spain; ^9^ Servicio de Neurología, Hospital General Universitario Gregorio Marañón/Red de Enfermedades Inflamatorias (REI) Madrid Spain; ^10^ Department of Analytical Chemistry, Faculty of Chemical Sciences Universidad Complutense de Madrid Madrid Spain; ^11^ Departamento de Neurología Hospital Universitario Quironsalud Madrid Madrid Spain

## Abstract

**Objective:**

An alteration in the composition of the intestinal microbiota has been observed in patients with multiple sclerosis (pwMS) with respect to healthy controls (HC). Microorganism‐derived metabolites such as short‐chain fatty acids (SCFA) have been suggested to play a role in the disease. Thus, to analyze the association of SCFA with clinical and radiological parameters of the disease and with those related to the inflammatory response of the immune system.

**Methods:**

Multicentric observational retrospective cross‐sectional study. In addition 161 pwMS and 130 HC were included. The following plasma SCFA were analyzed using liquid chromatography coupled to mass spectrometry: acetate (AA), propionate (PA) and butyrate (BA). Blood cell subpopulations and cytokine expression were analyzed by flow cytometry.

**Results:**

Plasma PA and PA/AA ratio was lower in pwMS than in HC (*P* = 0.0001, and *P* = 0.00005, respectively). PA/AA and BA/AA ratios were lower in pwMS with higher disability (*P* = 0.001, and *P* = 0.001, respectively). T2 lesion load inversely correlated with PA/AA (*r* = −0.353; *P* = 0.002) and BA/AA (*r* = −0.322; *P* = 0.005) ratios. Plasma PA/AA and/or BA/AA ratios negatively correlated with the following pro‐inflammatory cytokines producing cells: GM‐CSF+CD4+T, GM‐CSF+CD8+T, TNF‐alpha+CD4+T, TNF‐alpha+CD8+T, IFN‐gamma+CD4+T, IFN‐gamma+CD8+T, and TNF‐alpha+B cells.

**Interpretation:**

In MS, plasma PA/AA and BA/AA ratios are unbalanced, promoting an environment that could be boosting the mechanisms underlying the pathogenesis of the disease. Since we have found statistical significant associations with the EDSS and the number of T2 lesions, but not with the number of relapses or gadolinium enhancing lesions, PA/AA and BA/AA ratios could be more associated with those mechanisms of the disease related to the neurodegenerative processes than those related with the activity of the disease.

## Introduction

Multiple sclerosis (MS) is a chronic autoimmune disease of the central nervous system causing inflammation, demyelination, and neurodegeneration. Although its etiology is uncertain, both environmental and genetic risk factors are involved in its development. Gut microbiota has recently emerged as a likely contributor to MS.^1^ One of the most relevant studies in this area so far analyzed the gut microbiome of 576 MS patients, and 1,152 genetically unrelated household healthy controls (HC), observing significant differences in the proportion of the species described.^2^ Similarly, other studies have shown the depletion or enrichment of certain bacteria in MS compared with HC. Nevertheless, they are rarely concordant and it is difficult to identify a common pattern.^3^, ^4^, ^5^, ^6^, ^7^, ^8^, ^9^

On the other hand, microorganism‐derived metabolites such as short‐chain fatty acids (SCFAs) have been suggested to play a role in the microbiota–gut–brain axis.^10^ SCFAs are produced from the dietary fibers fermented by gut microorganisms in the large intestine, with the most abundant being butyrate, propionate, and acetate (95% of SCFAs).^11^ Functions associated with SCFAs include barrier function, immunoregulatory, and as an energy source for colonocytes (epithelial cells) in the colon. They are rapidly absorbed, and only about 5% of them are excreted in the feces, making them play a key role as immune and metabolic mediators.^12^ SCFAs would enter the systemic circulation and could directly impact the function and metabolism of peripheral organs and tissues, such as the liver, pancreas, adipocytes, immune system cells, and musculoskeletal tissue.^13^ The pathways for acetate (AA) production are common among different species of bacteria,^14^ while the pathways for propionate (PA) and butyrate (BA) are more conserved and substrate‐specific.^15^ Considering that different phylum, families, genus, and species are altered in MS patients,^11^ it is logical to think that the levels of SCFAs could be also modified in MS patients compared with a healthy population. Our previous results examining the plasma SCFAs in MS patients seemed to point to this condition.^16^, ^17^

In order to deep into the role of these metabolites, we analyze their association with different parameters of the disease, both clinical, radiological and those related to the inflammatory response of the immune system, in the largest cohort of untreated MS patients so far.

## Materials and Methods

### Study design

We conducted a multicentric observational retrospective cross‐sectional study, including samples from MS patients and healthy controls (HC). MS patients were recruited from the following Hospitals in Spain: Hospital General Universitario Gregorio Marañón, Hospital Universitario de Getafe, Hospital Universitario de Torrejón, Hospital Álvaro Cunqueiro, Hospital Universitari Vall d'Hebron, and Hospital Universitario Ramón y Cajal. HC matched by sex and age with the patients were recruited among the volunteered blood donors of Hospital Clínico San Carlos.

MS patients met the following inclusion criteria: (1) older than 18 years, (2) a diagnosis of relapsing–remitting multiple sclerosis (RRMS) according to 2010 or 2017 revised McDonald diagnostic criteria as appropriate,^18^, ^19^ (3) availability of stored peripheral blood mononuclear cells (PBMCs) and plasma samples, (4) free from any disease‐modifying treatment (DMTs) for the last 6 months or corticosteroids in the last 3 months previous to the sample collection, and (5) based on previous results of our group,^16^ we only recruited two subgroups of MS patients: those without disability, EDSS < 2 (named MS2), and those with severe disability, EDSS > 4 (named MS4).

Exclusion criteria for both, MS patients and HC, were (1) pregnant women and/or (2) MS patients with other concomitant neurological or autoimmune pathologies and HC having any relationship of consanguinity with MS patients or patients with other autoimmune diseases.

### Ethics statement

Informed consent was obtained from all subjects involved in this study, which was conducted in accordance with the Declaration of Helsinki. This study was approved by the local Ethic Committee of the Hospital Clínico San Carlos (Comité Ético de Investigación Clínica del Hospital Clínico San Carlos).

### Data and sample collection

All subjects involved in this study were included from June 2005 to May 2022. Data collection includes the following variables: (1) demographical variables: sex and age; (2) clinical variables at the sample collection: age at the disease onset, disease duration, EDSS score, MSSS (MS severity score),^20^ number of relapses since the disease onset; (3) radiological variables at the sample collection: number of T2 and gadolinium‐enhanced lesions at magnetic resonance imaging (MRI).

A whole blood sample obtained by venipuncture from every patient was collected in a cell preparation tube (CPT, BD vacutainer) for PBMCs and plasma isolation by Ficoll density gradient centrifugation. Plasma and PBMCs (aliquots of 5–10 × 10^6^ cells) samples were cryopreserved and eventually stored at −80°C and in liquid nitrogen (−196°C), respectively, until use.

### SCFAs levels determination

Plasma levels of the SCFAs: AA, PA, and BA were analyzed by liquid chromatography‐mass spectrometry in an 8,030 Shimadzu triple‐quadrupole mass spectrometer equipped with an ESI ionization source and operating in negative mode was used for determination of the selected metabolites, using the same methodology described in a previous study.^16^ Data were obtained and processed with LabSolution software.

### White blood cell population analysis

#### Labeling of surface antigens

Freshly thawed PBMC were resuspended in RPMI 1640 medium (Thermofisher Scientific, Waltham, MA), stained with the appropriate amounts of monoclonal antibodies (Table S1) for 30 min at 4°C in the dark, washed twice with PBS, and analyzed in a FACSCanto II flow cytometer (BD Biosciences).

#### In vitro stimulation and intracellular cytokine staining

Freshly thawed PBMC were resuspended in RPMI 1640 medium (Thermofisher Scientific, Waltham, MA) and stimulated with 50 ng/mL Phorbol 12‐myristate 13‐acetate (PMA) and 750 ng/mL Ionomycin (Sigma‐Aldrich, St. Louis, MO), in the presence of 2 μg/mL Brefeldin A and 2.1 μM Monensin (BD Biosciences), during 4 h. After incubation, PBMC were washed with PBS and stained for 30 min at 4°C in the dark with appropriate amounts of monoclonal antibodies recognizing the surface antigens (Table S1). Then, cells were washed with PBS, fixed, and permeabilized for 20 min at 4°C in the dark with Cytofix/Cytoperm Kit (BD Biosciences), washed twice with Perm/Wash solution (BD Biosciences), and stained intracellularly 30 min at 4°C in the dark with monoclonal antibodies recognizing the following cytokines: IFN‐gamma, TNF‐alpha, IL‐17, and IL‐10 (Table S1). After two washes, PBMC was analyzed in a FACSCanto II flow cytometer (BD Biosciences).

#### Flow cytometry

Cells were always analyzed within a maximum period of 1 h after staining. Mean autofluorescence values were set using appropriate negative isotype controls. Data analysis was performed using FACSDiva Software V.8.0 (BD Bio‐sciences). A gate including lymphocytes and monocytes and excluding debris and apoptotic cells was established; a minimum amount of 100.000 events were analyzed. The gating strategy to identify the different leukocyte populations is shown in Figure S1. We followed the strategy detailed below to identify the different subpopulations. CD4^+^ and CD8^+^ T cells were classified as naıve (CCR7^+^ CD45RO^−^), central memory (CM) (CCR7^+^ CD45RO^+^), effector memory (EM) (CCR7^−^ CD45RO^+^), and terminally differentiated (TD) (CCR7^−^ CD45RO‐). Regulatory CD4 T cells (Treg) were defined as CD3^+^ CD4^+^ CD25hi CD127^−^/low. CD56 NK cells were classified as NKT cells (CD3^+^ CD56dim), CD56dim NK cells (CD3^−^ CD56dim), and CD56bright NK cells (CD3^−^ CD56br). B cells were classified as naïve (CD19^+^ CD38dim CD27^−^), memory (CD19^+^ CD27dim CD38dim), plasmablasts (CD19^+^ CD27hi CD38hi), or transitional B cells (CD19^+^ CD27^−^ CD24hi CD38hi) cells. Monocytes were also studied, and their subsets were classified according to the expression of CD14 and CD16 (Mo1/classical: CD14hi CD16^−^; Mo2/intermediate: CD14hi CD16^+^; Mo3/nonclassical: CD14low CD16^+^). Dendritic cells (DCs) were defined as HLA‐DR^+^ CD3^−^ CD19^−^ CD56^−^ and differentiated into myeloid DCs (mDC: CD123^+^ CD11c^+^) and plasmacytoid DCs (pDC: CD123^+^ CD11^+^). Finally, we explored in CD4 and CD8 T cells the production of IFN‐gamma and TNF‐alpha, products of the Th1 response; IL‐17, a product of the Th17 response and GM‐CSF, which induces innate cell activation. Finally, we assessed B cells producing TNF‐alpha, an inflammatory cytokine and GM‐CSF, inducing innate cell activation. The percentage of every subset was expressed over total mononuclear cells. For every leukocyte subset, we recorded the total cell counts per mL of blood. They were calculated by measuring the total lymphocyte and monocyte numbers by a coulter counter, and the percentages of every subset over total mononuclear cells. We also recorded the values of every T, B, NK, and monocyte subset relative to total T, B, NK, and monocyte cells, respectively, to avoid bias due to B cell depletion.

### Statistical analysis

Categorical variables were expressed as percentages, normal numerical variables as mean ± standard deviation, and non‐normal as median (25th, 75th percentile). The association between/among categorical variables was analyzed using the chi‐square test, or Fisher's exact test when the value of the expected count less than 5 is more than 20%. For the quantitative variables, the means was compared using the test Student's *t*‐test or analysis of variance (ANOVA, for comparisons of more than two groups) or the Mann–Whitney U‐test, or the Kruskal–Wallis test (to compare the medians of several groups) in case the quantitative variables did not fit a normal distribution. The parametric Pearson coefficient or the nonparametric Spearman coefficient was applied to evaluate the correlation between two continuous quantitative variables. Since a correlation coefficient <0.3 or > −0.3 could be considered as negligible correlation,^21^ we only took into account those above or below these values, respectively. Subjects with missing data were omitted from the corresponding analyses. *P*‐values < 0.05 were referred to as statistically significant in the text. When necessary, the Bonferroni adjustment was carried out. Additionally, when it was required, logistic or linear regressions were performed to adjust *P*‐values for the appropriate factors. All analyses were conducted using SPSS for Windows (Ver. 21.0) software (SPSS Inc.), and plots were elaborated with Prism version 8.0 (GraphPad Prism, San Diego, CA, USA).

## Results

### Demographical, clinical, and radiological characteristics

We recruited 161 MS patients and 130 HC with the demographic, clinical, and radiological characteristics summarized in Table 1. We did not find any sex or age difference between MS patients and HC, whereas the MS4 age was statistically higher than the MS2 one.

**Table 1:** Demographic and clinical characteristics of the multiple sclerosis (MS) patients and healthy controls (HC) included in the study.

	MS (*n* = 161)	HC (*n* = 130)	*P*	MS2 (*n* = 113)	MS4 (*n* = 48)	*P*
Sex (male/female)	39/122	34/96	n.s.	25/88	14/34	n.s.
Age at sampling (years, mean ± SD)	40.0 ± 9.4	39.5 ± 9.3	n.s.	37.8 ± 9.0	44.9 ± 8.3	5.5 × 10^−6^
Age at MS onset (years, mean ± SD)	34.4 ± 14.5	‐	‐	35.6 ± 14.9	30.7 ± 12.5	n.s.
Disease duration (months, md (P25–P75))	81 (0–120)	‐	‐	24 (0–84)	132 (96–213)	3.8 × 10^−12^
EDSS score at sampling (md (P25–P75))	1.0 (0.0–5.0)	‐	‐	1.0 (0.0–1.5)	6.0 (5.0–6.5)	2.5 × 10^−23^
MSSS at sampling (md (P25–P75))	2.2 (0.7–5.3)	‐	‐	0.8 (0.5–2.4)	6.5 (5.5–7.9)	2.7 × 10^−21^
Relapses since MS onset (md (P25–P75))	2 (1–4)	‐	‐	2 (1–3)	7 (2–10)	1.8 × 10^−10^
Last DMT before sampling (*n* (%))						
None	102 (63.4%)	‐	‐	80 (70.8%)	21 (43.8%)	3.9 × 10^−5^
MET	48 (29.8%)	‐	‐	32 (28.3%)	19 (44.2%)	
HET	11 (6.8%)	‐	‐	1 (0.9%)	8 (16.7%)	
Gadolinium‐enhancing lesions (md (P25–P75))	0 (0–2)	‐	‐	0 (0–2)	0 (0–2)	n.s
T2 lesions at baseline (md (P25–P75))	23 (10–35)	‐	‐	22 (7, 36)	23 (10.5, 30)	n.s.

MS2: MS patients with EDSS < 2. MS4: MS patients with EDSS > 4. Continuous nonparametric variables are expressed as median (25th, 75th percentile) [md (P25–P75)] whereas parametric ones as mean ± standard deviation (m ± SD). Bonferroni correction (*P* < 0.005).

HET, high efficacy treatments (natalizumab, mitoxantrone, azathioprine); MET, moderate efficacy treatments (beta‐interferon, glatiramer acetate, dimethyl fumarate and teriflunomide); n.s., not significant.

### SCFA levels in MS patients and HC

**Figure 1:**
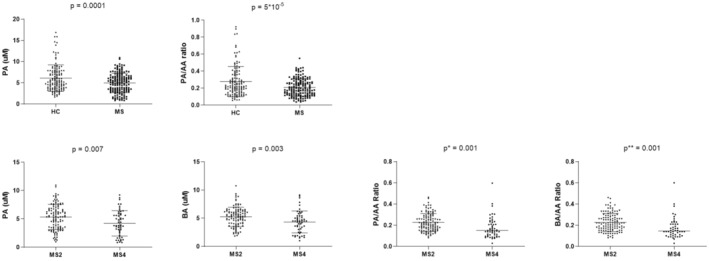
Significant comparisons of SCFA concentrations and their ratios in: (A) MS patients and HC. (B) Both groups of MS patients: MS2 (EDSS < 2) and MS4 (EDSS > 4). Significations with the two‐tailed *t*‐test are shown. *Adjusted *P*‐value for age using logistic regression = 0.0021. **Adjusted *P*‐value for age using logistic regression = 0.0025.

As depicted in Figure 1, plasma PA levels were lower in MS patients compared to HC (*P* = 0.0001), whereas no differences were found for AA or BA levels. To minimize the possible influence of conversion between the SCFAs, their uptake by peripheral organs and alterations during sample processing we analyzed SCFAs ratios. The PA/AA ratio was significantly lower (*P* = 0.00005) in MS patients compared with HC (Fig. 1).

When we compared both groups of MS patients, we found that those with higher EDSS score (MS4) showed significantly lower PA (*P* = 0.007) and BA (*P* = 0.003) levels than those with lower degrees of disability (MS2). The same results were obtained for PA/AA (*P* = 0.001) and BA/AA (*P* = 0.001) ratios (Fig. 1).

**Figure 2:**
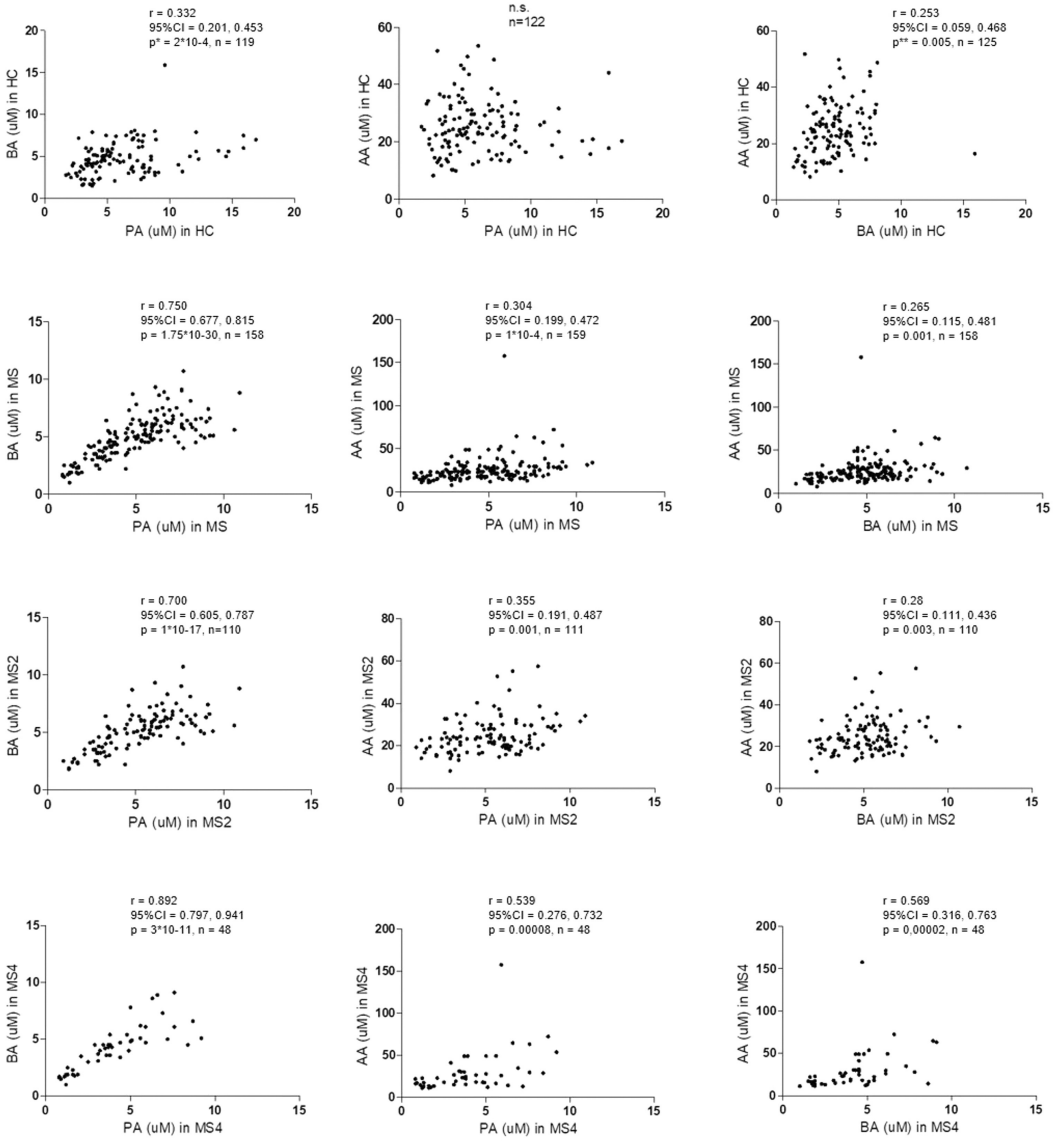
Correlation between SCFA in MS patients and HC and in both groups of MS patients (MS2: EDSS < 2; MS4: EDSS > 4). Correlations were assessed by using the Spearman's rank correlation coefficient (*r*); 95% CI, 95% confidence interval; n.s., not significant. *Adjusted *P*‐value for age using linear regression = 7.9 ×10–6. **Adjusted *P*‐value for age using linear regression = 0.02.

Moreover, correlation coefficients between BA‐PA and BA‐AA were lower among HC compared with MS patients (Fig. 2). Additionally, patients with higher EDSS scores showed higher correlation coefficients for BA‐PA, BA‐AA, and even for PA‐AA compared to those with low disability score. Correlation between BA and PA (*r* = 0.892, *P* = 3 × 10 × 11) found among patients with an EDSS > 4 (Fig. 2) was remarkably higher.

### SCFA level association with demographical variables in MS patients

We did not observe any significant difference in SCFA levels or their ratios among genders in MS patients or in HC (Table S2). Regarding age, only a significant correlation was found for PA (*r* = 0.249; *P* = 0.005) and PA/AA ratio (*r* = 0.264; *P* = 0.003) in HC, although both coefficients of correlation were under 0.3. We did not find any correlation with the age for any of the SCFA in MS patients.

### SCFA level association with clinical variables in MS patients

As shown in Table 2, we found the following significant associations after Bonferroni correction: AA positively correlated with the EDSS (*P* = 0.001), BA negatively correlated with MSSS (*P* = 0.008) and PA (*P* = 0.008) and BA (*P* = 0.009) negatively correlated with the number of relapses since the beginning of the disease (although with a negligible correlation coefficient). The PA/AA and BA/AA ratios confirmed a negative correlation with EDSS (*P* = 0.001 and *P* = 0.0001, respectively) and MSSS (*P* = 0.001 and *P* = 0.0001, respectively). Only the association of BA/AA ratio with EDSS (*r* = −0.303) and MSSS (*r* = −0.311) had a coefficient of correlation below −0.3 or above 0.3.

**Table 2:** Correlation between SCFA and their ratios with the clinical variables of MS patients.

	EDSS	MSSS	Relapses from MS onset
AA (μM)	*r* = 0.267	n.s.	n.s.
	*P* = 0.001		
	*n* = 151		
PA (μM)	n.s.	n.s.	*r* = −0.295
			*P* = 0.008
			*n* = 116
BA (μM)	n.s.	*r* = −0.211	*r* = −0.243
		*P* = 0.008	*P* = 0.009
		*n* = 157	*n* = 115
PA/AA	*r* = − 0.262	*r* = −0.266	*r* = −0.235
	*P* = 0.001	*P* = 0.001	*P* = 0.011
	*n* = 151	*n* = 150	*n* = 116
BA/AA	***r* = −0.303**	***r* = −0.311**	
	**95% CI = −0.439 to − 0.155**	**95% CI = −0.463 to −0.166**	*r* = −0.227
	***P* = 0.0001**	***P* = 0.0001**	*P* = 0.015
	***n* = 150**	***n* = 149**	*n* = 115

Correlations were assessed by using the Spearman's rank correlation coefficient (*r*). Bold values indicate both the statistically significant values after Bonferroni correction (*P* < 0.017) and correlation coefficients >0.3 or < −0.3.

95% CI, 95% confidence interval; n.s., not significant.

### SCFA level association with radiological variables in MS patients

**Figure 3:**
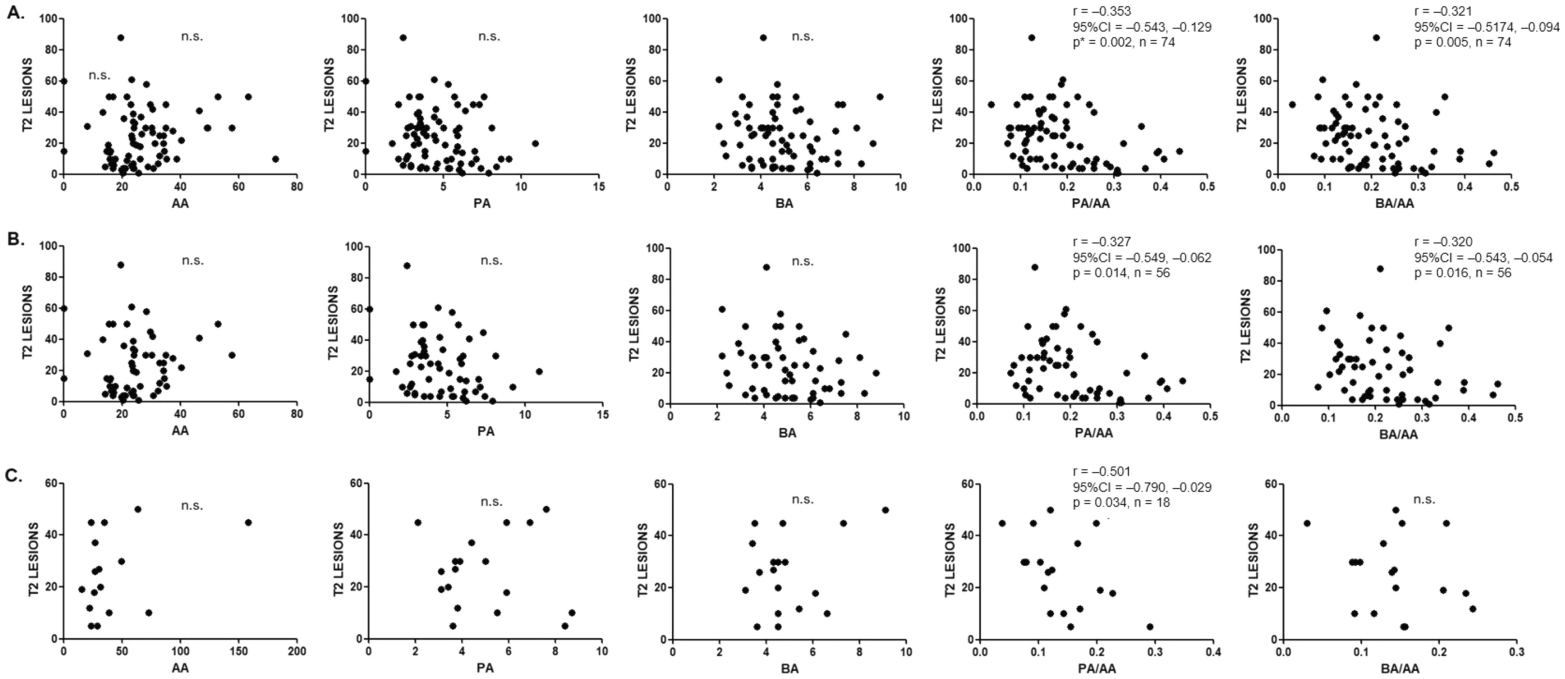
Correlations between SCFA concentrations and their ratios with T2 lesion loads: (A) MS patients. (B) MS2 patients (EDSS<2). (C) MS4 patients (EDSS>4). Correlations were assessed by using the Spearman's rank correlation coefficient (*r*); 95% CI, 95% confidence interval; n.s., not significant. *Adjusted *P*‐value for age using linear regression = 0.0017.

The number of T2 lesions on MRI was inversely correlated with the PA/AA (*r* = −0.353; *P* = 0.002) and BA/AA (*r* = −0.322; *P* = 0.005) ratios (Fig. 3A). When we analyzed the subgroups of MS patients, MS2 and MS4, both ratios were also significantly associated with the number of T2 lesions in MS2 patients (*r* = −0.327; *P* = 0.01 for PA/AA and *r* = −0.320; *P* = 0.02 for BA/AA), but only PA/AA was significantly correlated in patients with higher EDSS (*r* = −0.501; *P* = 0.03) (Fig. 3B,C). When we compared the SCFA levels above and below the median value of the T2 lesion load (23), we found again that the PA/AA (*P* = 0.007) and the BA/AA (*P* = 0.01) ratios were significantly decreased in those MS patients with a higher number of T2 lesions (Table 3). Regarding the number or the presence of gadolinium‐enhancing lesions, we did not find any significant association.

**Table 3:** SCFA levels and their ratios in MS patients with T2 lesions above and below the median value.

	T2 lesions < 23	T2 lesions > 23	*P*
AA (μM): md (P25–P75)	23.6 (19.9–31.1)	28.1 (23.2–35.5)	n.s.
PA (μM): md (P25–P75)	5.1 (3.5–6.2)	3.8 (3.3–5.7)	n.s.
BA (μM): md (P25–P75)	5.1 (4.1–6.3)	4.5 (3.7–5.6)	n.s.
PA/AA: md (P25–P75)	0.21 (0.13–0.27)	0.14 (0.12–0.18)	0.007
BA/AA: md (P25–P75)	0.21 (0.15–0.26)	0.15 (0.12–0.21)	0.01*

Continuous nonparametric variables are expressed as median (25th, 75th percentile) [md (P25–P75)]. Significations with the Mann–Whitney *U*‐test are shown.

n.s., not significant.

* Adjusted *P*‐value for age using logistic regression = 0.009.

### SCFA levels and inflammatory profile in MS patients and HC

Correlations for the SCFA and their ratios with the different subpopulations of cells analyzed in all the groups can be consulted in Table S3, while correlations for the SCFA and their ratios with the expression of intracellular cytokines in all the groups are shown in Table S4.

**Figure 4:**
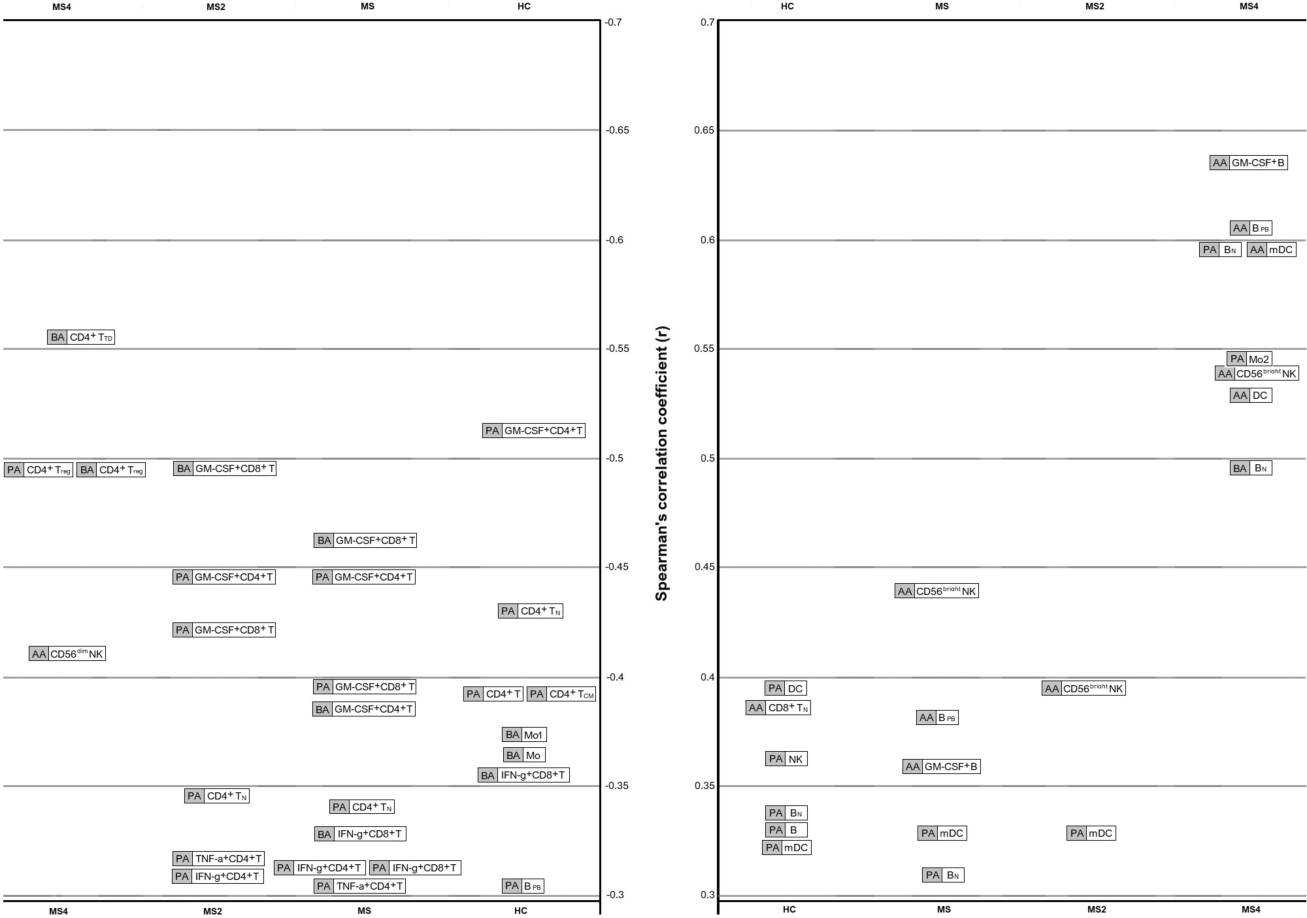
Graphic with the main correlations (only those with a Spearman's rank correlation coefficient >0.3 or < −0.3) between the SCFA, percentages of cell subpopulations and the expression of cytokines in HC, MS patients and both subgroups of MS patients (MS2 and MS4). AA, acetate; B, B lymphocytes; BA, butyrate; CD4^+^ T, CD4^+^ T lymphocytes; CD8^+^ T, CD8^+^ T lymphocytes; CM, central memory; DC, dendritic cells; GM‐CSF, granulocyte‐macrophage colony‐stimulating factor; IFN‐g, interferon gamma; mDC, myeloid dendritic cells; Mo, monocytes; N, naïve; NK, natural killer cells; PA, propionate; PB, plasmablasts; reg: regulators; TD, terminally diferentiated; TNF‐a, tumor necrosis factor alpha.

Opposite results were found in relation to the cytokine‐producing cells (Fig. 4): While PA and BA were negatively correlated with GM‐CSF, IFN‐gamma, and TNF‐alpha producing cells, AA was positively correlated with GM‐CSF producing cells. Thus, when we considered the PA/AA and BA/AA ratios, we mainly found negative correlations with different pro‐inflammatory cytokine‐producing cells (Table 4).

**Table 4:** Significant correlations between PA/AA and BA/AA ratios with the different percentages of cell populations in MS patients.

Positive correlations		Negative correlations	
PA/AA	BA/AA	PA/AA	BA/AA
CD56^dim^ NK (*r* = 0.437; 95% CI = 0.272–0.576; *P* = 3 × 10–7)	CD56^dim^ NK (*r* = 0.416; 95% CI = 0.251–0.561; *P* = 1 × 10–6)	GM‐CSF+CD4+T (*r* = −0.473; 95% CI = −0.648 to −0.273; *P* = 5 × 10–6)	GM‐CSF+CD8+T (*r* = −0.453; 95% CI = −0.607 to −0.259; *P* = 1 × 10–5)
		GM‐CSF+CD8+T (*r* = −0.416; 95% CI = −0.570 to −0.248; *P* = 7 × 10–5)	GM‐CSF+CD4+T (*r* = −0.426; 95% CI = −0.590 to −0.242; *P* = 5 × 10–5)
		TNF‐α+CD4+T (*r* = −0.383; 95% CI = −0.564 to −0.171; *P* = 0.0002)	CD56^bright^ NK (*r* = −0.338; 95% CI = −0.496 to −0.165; *P* = 0.0002)
		TNF‐α+CD8+T (*r* = −0.358; 95% CI = −0.534 to −0.164; *P* = 0.0005)	IFN‐γ+CD8+T (*r* = −0.315; 95% CI = −0.497 to −0.075; *P* = 0.002)
		IFN‐γ+CD8+T (*r* = −0.354; 95% CI = −0.519 to −0.158; *P* = 0.0009)	TNF‐α+CD8+T (*r* = −0.324; 95% CI = −0.498 to −0.136; *P* = 0.002)
		IFN‐γ+CD4+T (*r* = −0.333; 95% CI = −0.520 to −0.144; *P* = 0.001)	TNF‐α+CD4+T (*r* = −0.309; 95% CI = −0.489 to −0.107; *P* = 0.003)
		CD4^+^ T (*r* = −0.318; 95% CI = −0.473 to −0.152; *P* = 0.0001)	BPB (*r* = −0.302; 95% CI = −0.465 to −0.142; *P* = 0.001)
		TNF‐α+B (*r* = −0.309; 95% CI = −0.492 to −0.113; *P* = 0.003)	
		CD4+TN (*r* = −0.305; 95% CI = −0.462 to −0.141; *P* = 0.0005)	

Correlations were assessed by using the Spearman's rank correlation coefficient (*r*); only significant correlations with *r* > 0.3 or *r* < −0.3 are shown. Bonferroni correction (*P* < 0.00128).

95% CI, 95% confidence interval; AA, acetate; B, B lymphocytes; BA, butyrate; CD4^+^ T, CD4^+^ T lymphocytes; CD8^+^ T, CD8^+^ T lymphocytes; GM‐CSF, granulocyte‐macrophage colony‐stimulating factor; IFN‐γ, interferon gamma; N, naïve; NK, natural killer cells; PA, propionate; PB, plasmablasts; TNF‐a, tumor necrosis factor alpha.

## Discussion

MS is a complex disease, with different clinical manifestations depending on each patient. However, all of them suffer a process of increase in their disability whose causes are not well understood. In this study, we found lower levels of PA and the PA/AA ratio in MS patients compared with HC. In addition, BA and PA concentrations together with the BA/AA and PA/AA ratios were remarkably lower in patients with higher disability. Furthermore, PA/AA and BA/AA ratios were significantly lower among patients with a higher number of T2 lesions. Eventually, leukocyte subpopulation study results point out that whereas PA and BA seem to have an anti‐inflammatory profile, AA would have a pro‐inflammatory role, showing that the PA/AA and BA/AA ratios have a good correlation with the inflammatory environment.^17^

Only a few articles have been published measuring AA, BA, and/or PA in the serum or plasma of MS patients compared with those of HC. Our results are in accordance to some previous studies,^22^, ^23^, ^24^ but not with others.^25^, ^26^ These discrepancies could be due to the low number of samples analyzed and the heterogeneous cohorts of MS patients recruited in these studies, with a different proportion of patients under different kinds of treatments. This last aspect is crucial as showed Jangi et al.^3^ when they found that relative abundances of genera in the fecal microbiota were significantly altered between untreated and treated MS patients. Thus, here we have conducted this study including the largest cohort of untreated MS patients so far.

The results of the current study go in the same direction that in the previous ones performed by our group.^16^, ^17^, ^27^ As in previous studies, we recruited MS patients without disability (EDSS < 2; named MS2), and those with severe disability (EDSS > 4; named MS4) to compare the different variables between both subgroups. Again, significant lower levels of PA and the PA/AA ratio in MS patients compared with HC, and BA and PA concentrations and the BA/AA and PA/AA ratios were also lower in patients with higher disability. However, this is the first time that we have studied the possible association of SCFA with the T2 lesion load on brain MRI. As we have shown, the PA/AA and BA/AA ratios were significantly lower among patients with a higher number of T2 lesions. This result could not be a surprise since previous works have described a correlation, although moderate, between the T2 lesion load and EDSS (*r* = 0.53; *P* < 0.0001).^28^ Therefore, we could suspect that if the PA/AA and BA/AA ratios were lower in patients with a higher EDSS, they would also be lower in patients with a higher T2 lesion load, as we have finally found (see Table 3). But what it is more remarkable is that we have also found significant correlations between PA/AA (*r* = −0.353; *P* = 0.002) and BA/AA (*r* = −0.322; *P* = 0.005) ratios with T2 lesion load in MS patients (Fig. 3). Thus, the balance between these SCFA seems to be associated with the brain injury responsible, at least in part, of the disability of the disease.

But, how could the SCFA exert their effect, contributing to the appearance of new T2 lesions? SCFA have the potential to interact with most of the cells of the immune system through passive diffusion and carrier‐mediated absorption through solute transporters.^29^ As shown in Figure 4, we found different results for AA in comparison with PA and BA, being the first more associated with a pro‐inflammatory profile. In MS patients, we found significant correlations between AA and the GM‐CSF‐producing B cells (*r* = 0.357; *P* = 0.0006), B cell plasmablasts (*r* = 0.377; *P* = 0.00001) and CD56^bright^ NK cells (*r* = 0.443; *P* = 0.0000004). GM‐CSF‐producing B cells promote an inflammatory phenotype of myeloid cells and is abnormally increased in patients with MS,^30^ plasmablasts have been identified as the main effector B cell population involved in ongoing active inflammation in MS patients,^31^ and CD56^bright^ NK cells, in general, are more potent cytokine and chemokine producers, as they secrete high levels of cytokines, such as IFN‐gamma and TNF‐alpha upon activation^32^; although contradictory findings have been reported in both MS and EAE, different experiments have shown that CD56^bright^ NK cells accumulate in inflammatory lesions and, in the appropriate cytokine environment, can engage with CD14^+^ monocytes in a reciprocal activatory fashion, amplifying the inflammatory response.^33^ Meanwhile, PA and BA showed negative correlations with GM‐CSF, IFN‐gamma, and TNF‐alpha producing cells in MS patients (Fig. 4). Thus, when we considered the PA/AA and BA/AA ratios, we mainly found negative correlations with different pro‐inflammatory cytokine‐producing cells (Table 4). Therefore, in MS patients, PA/AA and BA/AA ratios seem to be unbalanced in comparison with HC, promoting a pro‐inflammatory environment that could be boosting the mechanisms underlying the pathogenesis of the disease, finally reflected in a higher T2 lesion load and a higher EDSS. It is noteworthy that the highest correlations were found in MS4 subgroup (see Tables S3 and S4). Furthermore, in this subgroup we also found the highest correlation coefficients between the three SCFA analyzed (Fig. 2). A possible explanation of the evolution of these coefficients of correlation (*r* in HC < *r* in MS2 < *r* in MS4) could be a loss of diversity of bacterial species in MS. This loss of diversity would increase along the evolution of the disease, explaining the highest correlation coefficients found in MS patients with higher disability. However, more studies are needed to better understand this result, since contradictory findings have been published in relation to the possible loss of diversity in MS.^34^

Increasing the PA/AA and BA/AA ratios in MS patients to values similar to those found in HC could be crucial to reduce the inflammatory environment and to ameliorate the course of the disease. Currently, there are few studies that have analyzed the effects of PA or BA supplementation in MS patients or other inflammatory diseases. Duscha et al. supplemented a small cohort of MS patients with PA during 14 days; authors detected an increase in both the number and functionality of T reg cells, a decrease in the relapse rate and a stabilization of disability.^23^ Another study explored, in obese and non‐obese MS patients, PA concentrations as well as peripheral Th17 and Treg frequencies before and after 90 days of daily PA intake; they concluded that the administration of PA helped to restore the imbalance of T reg/Th17 cells.^35^ A 60‐day PA supplementation study was performed in patients with end‐stage renal disease, who suffer from a progressively increasing low‐grade systemic inflammation; results show a significant reduction of inflammatory marker C‐reactive protein under PA supplementation, and an expansion of circulating Tregs.^36^ Overall, these studies have shown promising results in the reduction of different inflammatory makers. Taking them together with the results of the current study, we could suggest to perform PA or BA supplementation studies monitoring PA/AA and BA/AA ratios. Furthermore, it would be very interesting to know the possible prognostic value of these ratios at the beginning of the disease and to evaluate the usefulness of PA or BA supplementation in these recently diagnosed patients to reduce the inflammatory environment since the first stages of the disease.

In conclusion, this study has found that the different SCFA could play different roles in the inflammatory process. While AA seems to be associated with pro‐inflammatory cell subsets and cytokines, PA and BA are more related to an anti‐inflammatory profile. PA/AA ratio is not only decreased in MS patients compared with HC, but together with BA/AA ratio, are remarkably lower in MS patients with higher EDSS. Finally, both ratios were statistically significantly lower in MS patients with a higher T2 lesion load. These results suggest that the unbalance in the SCFA of MS patients could stimulate a low‐grade inflammatory environment that could be enhancing the mechanisms involved in the pathogenesis of the disease. Since we have found statistical significant associations with the EDSS and the number of T2 lesions, but not with the number of relapses or gadolinium‐enhancing lesions, PA/AA and BA/AA ratios could be more associated with those mechanisms of the disease related to the neurodegenerative processes than those related with the activity of the disease.

## Author Contributions

MIDM and DLM prepared the samples, made the statistical analysis, discussed and interpreted findings, and revised the manuscript critically. MAGM processed the samples and revised the manuscript critically. AMM, EGC, and JLLG prepared the samples for LC‐MSMS analysis and performed such analysis. LMV, LCF, NV, YA, BP, XM, MC, ICP, IGS, MLMG, JMGD, and RA provided unique reagents, discussed and interpreted findings, and revised the manuscript critically. RAL contributed to the design of the study, guided the progress of the study, and wrote the manuscript. All authors read and approved the final manuscript and have agreed both to be personally accountable for the author's own contributions and to ensure that questions related to the accuracy or integrity of any part of the work, even ones in which the author was not personally involved, are appropriately investigated, resolved, and the resolution documented in the literature.

## Funding Information

This work was financially supported by Ministerio de Ciencia e Innovación (Proyectos de generación de conocimiento)‐Fondo Europeo de Desarrollo Regional (Feder) (PID2021‐126041OB‐I00) and “Fundación LAIR”.

## Conflict of Interest

Noelia Villarrubia, Yolanda Aladro, Ignacio Casanova‐Peño, Estefanía García‐Calvo, Machuca‐Marcos, Andrés, Jose Luis Luque‐García, María Ángel García‐Martínez, Daniel López‐Mecández, and María Inmaculada Domínguez‐Mozo: nothing to disclose. Luisa María Villar: has served at scientific advisory boards, participated in meetings sponsored by, received speaking honoraria or travel funding or research grants from Roche, Sanofi, Merck, Biogen, Bristol Myers, and Novartis. Lucienne Costa‐Frossard: reports compensation for consulting services and speaker honoraria from Biogen, Bristol Myers Squibb, Janssen, Merck‐Serono, Novartis, Sanofi, Roche, and Teva. Belén Pilo: has received speaker honoraria by Novartis and Almirall, travel honoraria by Merck and training honoraria by Sanofi and Merck. Xavier Montalbán: speaking honoraria and travel expenses for scientific meetings, has been a steering committee member of clinical trials or participated in advisory boards of clinical trials in the past 3 years with Actelion, Alexion, Biogen, Celgene, EMD Serono, Genzyme, Immunic, Medday, Merck, Mylan, Novartis, Roche, Sanofi‐Genzyme, and Teva Pharmaceutical. Manuel Comabella: compensation for consulting services and speaking honoraria from Bayer Schering Pharma, Merk Serono, Biogen‐Idec, Teva Pharmaceuticals, Sanofi‐Aventis, Genzyme, and Bristol‐Myers Squibband Novartis. Inés González‐Suárez: reports compensation for consulting services and speaker honoraria from Biogen, Janssen, Merck‐Serono, Novartis, Sanofi, and Roche. María Luisa Martínez‐Ginés: has received compensation for consulting services and speaking fees from Merck, Biogen, Novartis, Sanofi‐Genzyme, Almirall, ROCHE, BMS, and TEVA. Jose Manuel García‐Domínguez: has received speaker honoraria, research grants or advisor invitations from Bristol‐Myers‐Squibb, Johnson and Johnson, Biogen, Sanofi, Almirall, Merck, Roche, Zenas Pharma, and Novartis. Rafael Arroyo: has been a speaker or has participated in the advisory board of Novartis, Teva, Roche, Bristol, Janssem, Biogen, Merck, and Sanofi‐Genzyme. Roberto Álvarez‐Lafuente: has received support for attending meetings from Biogen, Merck, Novartis, and Sanofi‐Genzyme.

## Supporting information


**Figure S1.** Total events were first gated to exclude debris and apoptotic cells (all set of figures: A–D; gate P1) and then gated for doublet discrimination (all set of figures: A– D; gate P2). Cells were further analyzed to identify leukocytes (all set of figures: A– D; gate P3) for their CD45 staining, including monocytes (C, gate Mon) and lymphocytes (C, gate Lymph). (A). CD4^+^ and CD8^+^ T lymphocytes cell subpopulations. (B) NK and B cell subpopulations. (C) Monocytes and dendritic cell subpopulations. (D) intracellular cytokine‐producing B and T lymphocytes. CM, central memory; EM, effector memory; FSC‐A, forward scatter‐area; FSC‐H, forward scatter‐height; Mem, memory; NK++, NK bright cells; PB, plasmablasts; REG, regulatory; SSC, side scatter; TD, terminally differentiated; Trans B, transitional B cells.

**Table S1.** List of antibodies used for flow cytometry.

**Table S2.** Comparison between SCFA levels and their ratios among genders in MS patients and HC.

**Table S3.** Correlations for the SCFA and their ratios with the different subpopulations of cells in MS patients and controls.

**Table S4.** Correlations for the SCFA and their ratios with the expression of intracellular cytokines in MS patients and controls.

## Data Availability

The original contributions presented in the study are included in the article. Further inquiries can be directed to the corresponding author.
