# Supplementary figures and images for: Short‐chain fatty acids in multiple sclerosis: Associated with disability, number of T2 lesions, and inflammatory profile
Source: Ann Clin Transl Neurol. 2025 Mar 3;12(3):478–90. doi: 10.1002/acn3.52259 (PMC11920722; doi:10.1002/acn3.52259)

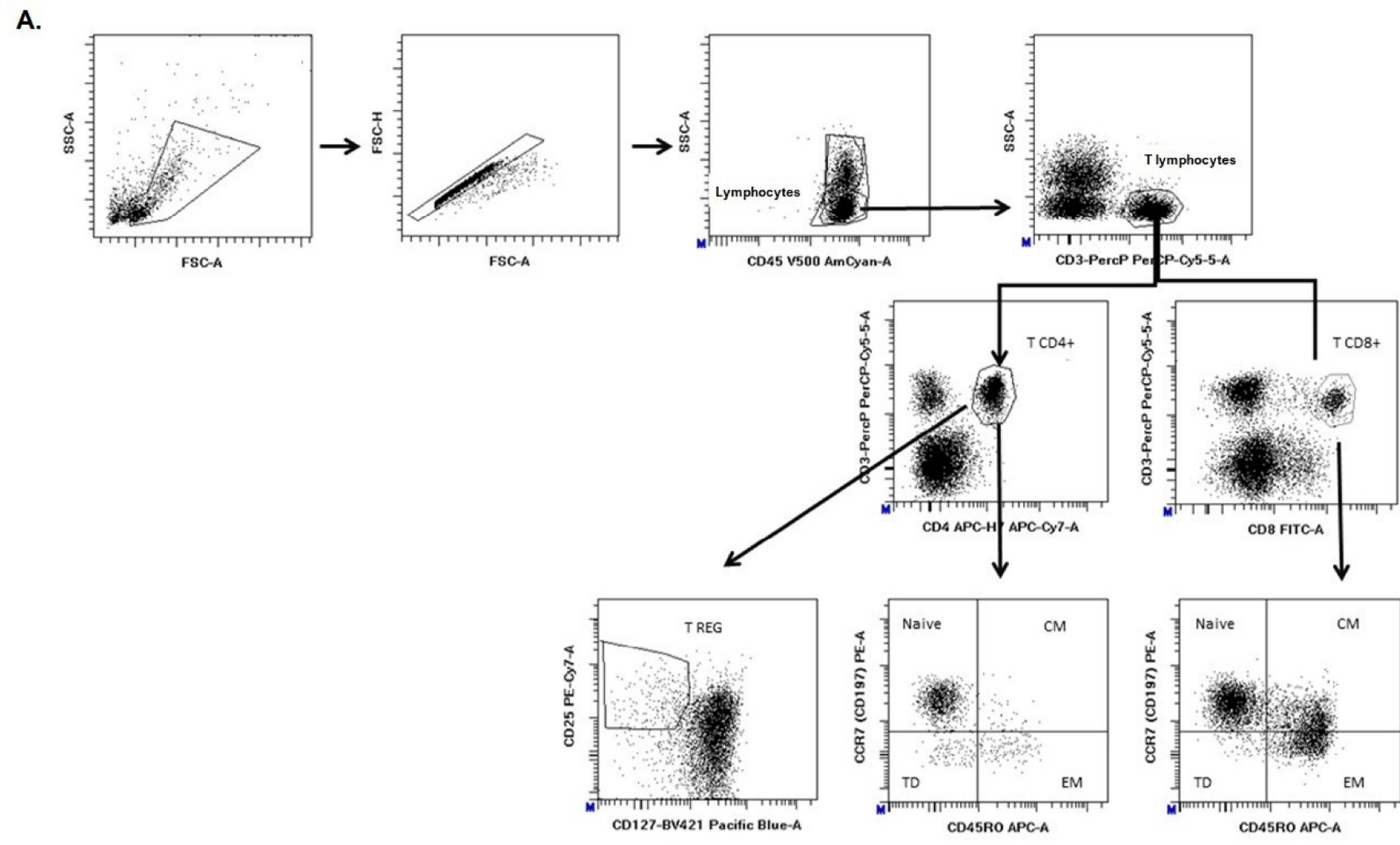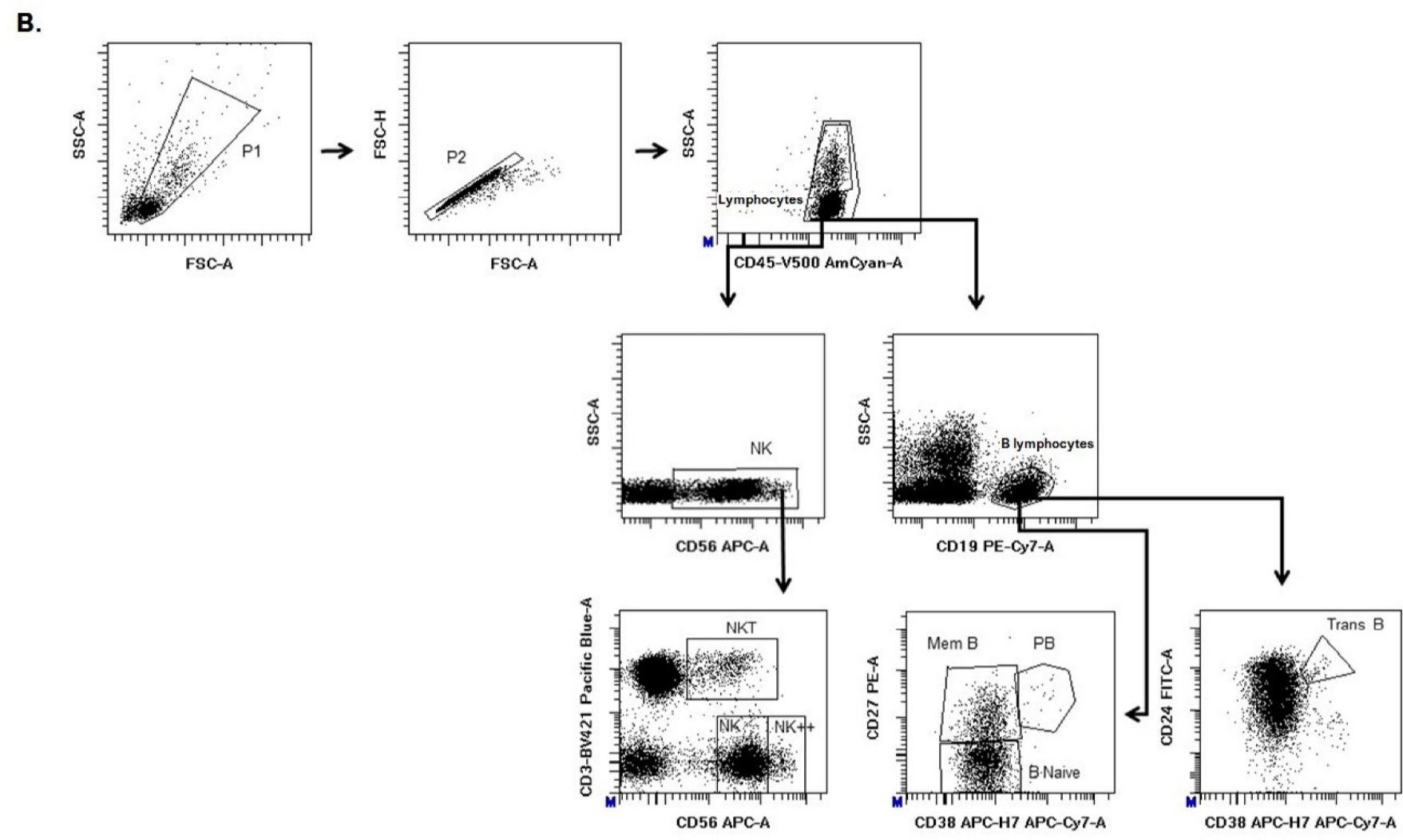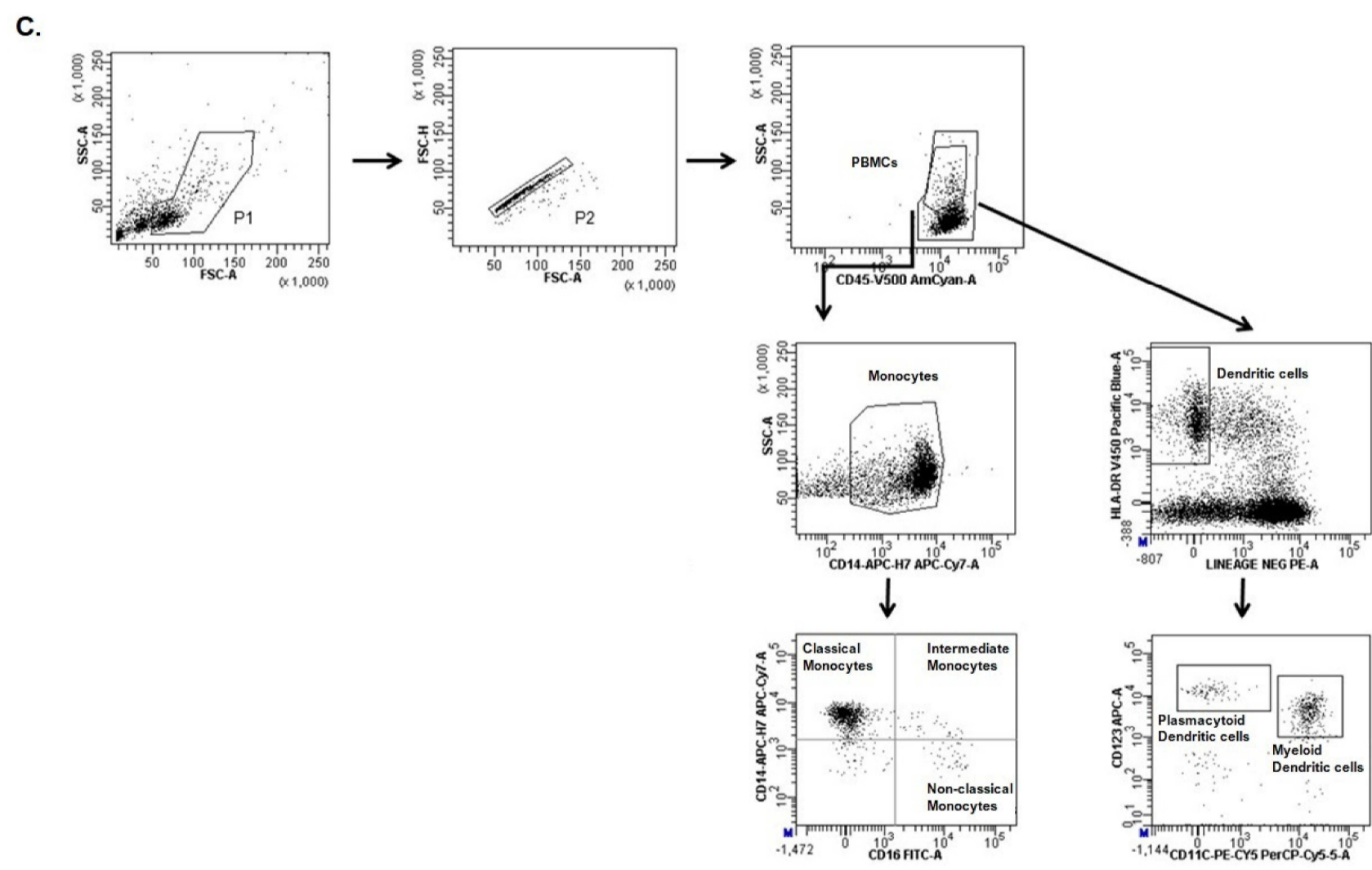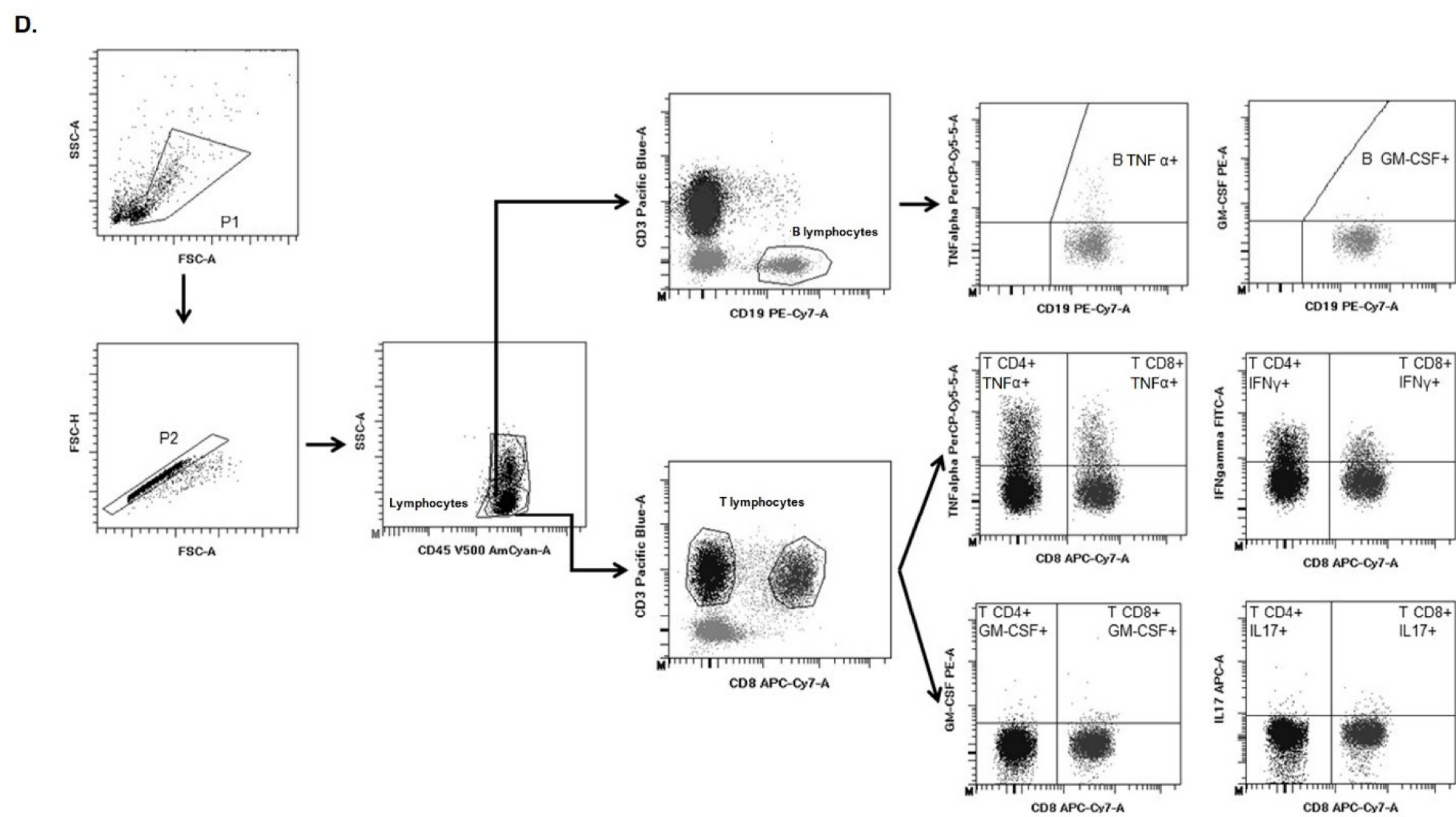

Supplement: Supplementary file 1 — Figure S1. Total events were first gated to exclude debris and apoptotic cells (all set of figures: A–D; gate P1) and then gated for doublet discrimination (all set of figures: A– D; gate P2). Cells were further analyzed to identify leukocytes (all set of figures: A– D; gate P3) for their CD45 staining, including monocytes (C, gate Mon) and lymphocytes (C, gate Lymph). (A). CD4+ and CD8+ T lymphocytes cell subpopulations. (B) NK and B cell subpopulations. (C) Monocytes and dendritic cell subpopulations. (D) intracellular cytokine‐producing B and T lymphocytes. CM, central memory; EM, effector memory; FSC‐A, forward scatter‐area; FSC‐H, forward scatter‐height; Mem, memory; NK++, NK bright cells; PB, plasmablasts; REG, regulatory; SSC, side scatter; TD, terminally differentiated; Trans B, transitional B cells. [file ACN3-12-478-s003.pdf]
