# Supplementary material for: Short‐chain fatty acids in multiple sclerosis: Associated with disability, number of T2 lesions, and inflammatory profile
Source: Ann Clin Transl Neurol. 2025 Mar 3;12(3):478–90. doi: 10.1002/acn3.52259 (PMC11920722; doi:10.1002/acn3.52259)
Supplement: Supplementary file 2 — Table S1. List of antibodies used for flow cytometry. [file ACN3-12-478-s004.docx]

| **Supplementary Table 1.** List of antibodies used for flow cytometry. | |  |
| --- | --- | --- |
|  |  |  |
|  |  |  |
| **Fluorochromes** | **Specificity** | **Supplier** |
| FTIC | CD8; CD24; CD16; IFNgamma | Becton Dickinson |
| PE | CCR7; CD27; CD3; CD56; CD19; GM-CSF | Becton Dickinson |
| PercP | CD3 | Becton Dickinson |
| PE-Cy7 | CD25; CD19 | Becton Dickinson |
| PE-Cy5 | CD11c | Becton Dickinson |
| PerCP-Cy5.5 | TNFα | Becton Dickinson |
| APC | CD45RO; CD56; CD123; IL-17 | Becton Dickinson and R&D Systems (IL-17) |
| APC-H7 | CD4; CD38; CD14; CD8 | Becton Dickinson |
| BV421 | CD127; CD3 | Becton Dickinson |
| V450 | HLA-DR | Becton Dickinson |
| V500 | CD45 | Becton Dickinson |
