# Supplementary material for: Short‐chain fatty acids in multiple sclerosis: Associated with disability, number of T2 lesions, and inflammatory profile
Source: Ann Clin Transl Neurol. 2025 Mar 3;12(3):478–90. doi: 10.1002/acn3.52259 (PMC11920722; doi:10.1002/acn3.52259)
Supplement: Supplementary file 3 — Table S2. Comparison between SCFA levels and their ratios among genders in MS patients and HC. [file ACN3-12-478-s005.docx]

**Supplementary Table 2.** Comparison between SCFA levels and their ratios among genders in MS patients and HC.

| **HC** |  | **MALE** | | **FEMALE** | | **p*** |
| --- | --- | --- | --- | --- | --- | --- |
|  |  | **Mean±SD** | **n** | **Mean±SD** | **n** |  |
|  | AA (uM) | 22,70±10,73 | 33 | 26,21±9,07 | 97 | n.s |
|  | PA (uM) | 6,45±3,36 | 32 | 6,07±2,99 | 93 | n.s |
|  | BA (uM) | 4,2±1,54 | 31 | 4,97±2,02 | 91 | n.s |
|  | PA/AA | 0,31±0,10 | 32 | 0,26±0,18 | 93 | n.s |
|  | BA/AA | 0,22±0,10 | 31 | 0,21±0,18 | 91 | n.s |
|  |  |  |  |  |  |  |
|  |  |  |  |  |  |  |
| **MS** |  | **MALE** | | **FEMALE** | | **p*** |
|  |  | **Mean±SD** | **n** | **Mean±SD** | **n** |  |
|  | AA (uM) | 26,12±12,48 | 42 | 26,11±15,83 | 117 | n.s |
|  | PA (uM) | 4,72±2,18 | 42 | 4,91±2,36 | 117 | n.s |
|  | BA (uM) | 4,98±1,83 | 41 | 4,88±1,87 | 117 | n.s |
|  | PA/AA | 0,20±0,09 | 42 | 0,20±0,10 | 117 | n.s |
|  | BA/AA | 0,21±0,09 | 41 | 0,20±0,09 | 117 | n.s |
|  |  |  |  |  |  |  |
|  |  |  |  |  |  |  |
| **MS2** |  | **MALE** | | **FEMALE** | | **p*** |
|  |  | **Mean±SD** | **n** | **Mean±SD** | **n** |  |
|  | AA (uM) | 25,31±9,85 | 23 | 24,21±7,18 | 88 | n.s |
|  | PA (uM) | 5,15±2,04 | 23 | 5,21±2,31 | 88 | n.s |
|  | BA (uM) | 5,53±1,70 | 22 | 5,10±1,73 | 88 | n.s |
|  | PA/AA | 0,21±0,88 | 23 | 0,22±0,09 | 88 | n.s |
|  | BA/AA | 0,23±0,09 | 22 | 0,22±0,08 | 88 | n.s |
|  |  |  |  |  |  |  |
|  |  |  |  |  |  |  |
| **MS4** |  | **MALE** | | **FEMALE** | | **p*** |
|  |  | **Mean±SD** | **n** | **Mean±SD** | **n** |  |
|  | AA (uM) | 27,11±15,30 | 19 | 31,87±28,84 | 29 | n.s |
|  | PA (uM) | 4,19±2,28 | 19 | 4,01±2,32 | 29 | n.s |
|  | BA (uM) | 4,33±1,80 | 19 | 4,23±2,14 | 29 | n.s |
|  | PA/AA | 0,17±0,10 | 19 | 0,16±0,10 | 29 | n.s |
|  | BA/AA | 0,18±0,09 | 19 | 0,17±0,10 | 29 | n.s |
|  |  |  |  |  |  |  |
| *Two-tailed t-test (n.s.: not significant). | | |  |  |  |  |
