# Supplementary material for: Short‐chain fatty acids in multiple sclerosis: Associated with disability, number of T2 lesions, and inflammatory profile
Source: Ann Clin Transl Neurol. 2025 Mar 3;12(3):478–90. doi: 10.1002/acn3.52259 (PMC11920722; doi:10.1002/acn3.52259)
Supplement: Supplementary file 4 — Table S3. Correlations for the SCFA and their ratios with the different subpopulations of cells in MS patients and controls. [file ACN3-12-478-s001.docx]

**Supplementary Table 3.** Correlations for the SCFA and their ratios with the different subpopulations of cells in MS patients and controls.

1. *CD4 T lymphocytes*

| **HC** |  | **AA** | **PA** | **BA** | **PA/AA** | **BA/AA** |
| --- | --- | --- | --- | --- | --- | --- |
|  | **T cells** | n.s. | n.s. | n.s. | r = - 0.276 ; p = 0.02; n = 75 | n.s. |
|  | **CD4 T cells** | n.s. | r = - 0.383 ; p = 0.0003 ; n = 84 | n.s. | r = - 0.378 ; p = 0.0004 ; n = 84 | n.s. |
|  | **reg CD4 T cells** | n.s. | n.s. | n.s. | r = - 0.244 ; p = 0.03; n = 84 | n.s. |
|  | **naive CD4 T cells** | r = 0.279 ; p = 0.01; n = 84 | r = - 0.430 ; p = 4*10-5 ; n = 84 | n.s. | r = - 0.477 ; p = 4*10-6 ; n = 84 | n.s. |
|  | **CM CD4 T cells** | n.s. | r = - 0.385 ; p = 3*10-4 ; n = 84 | n.s. | r = - 0.323 ; p = 0.003 ; n = 84 | n.s. |
|  | **TD CD4 T cells** | n.s. | n.s. | n.s. | n.s. | n.s. |
|  | **EM CD4 T cells** | n.s. | n.s. | r = 0.247 ; p = 0.03 ; n = 79 | n.s. | r = 0.260 ; p = 0.02 ; n = 79 |
|  |  |  |  |  |  |  |
|  |  |  |  |  |  |  |
| **MS** |  | **AA** | **PA** | **BA** | **PA/AA** | **BA/AA** |
|  | **T cells** | n.s. | r = - 0.265 ; p = 0.006 ; n = 106 | r = - 0.198 ; p = 0.04 ; n = 105 | r = - 0.211 ; p = 0.03 ; n = 106 | n.s. |
|  | **CD4 T cells** | n.s. | r = - 0.264 ; p = 0.002 ; n = 133 | r = - 0.211 ; p = 0.02 ; n = 132 | r = - 0.318 ; p = 0.0001 ; n = 133 | r = - 0.234 ; p = 0.007 ; n = 132 |
|  | **reg CD4 T cells** | n.s. | r = - 0.239; p = 0.007 ; n = 128 | r = - 0.263 ; p = 0.001 ; n = 127 | n.s. | n.s. |
|  | **naive CD4 T cells** | n.s. | r = - 0.338 ; p = 0.0001 ; n = 125 | r = - 0.255 ; p = 0.004 ; n = 124 | r = - 0.305 ; p = 0.0005 ; n = 125 | r = - 0.181 ; p = 0.044 ; n = 124 |
|  | **CM CD4 T cells** | n.s. | r = - 0.222 ; p = 0.013 ; n = 125 | r = - 0.201 ; p = 0.025 ; n = 124 | r = - 0.259 ; p = 0.004 ; n = 125 | r = - 0.204 ; p = 0.023 ; n = 124 |
|  | **TD CD4 T cells** | n.s. | r = - 0.179 ; p = 0.046 ; n = 125 | n.s. | n.s. | n.s. |
|  | **EM CD4 T cells** | n.s. | n.s. | n.s. | n.s. | n.s. |
|  |  |  |  |  |  |  |
|  |  |  |  |  |  |  |
| **MS2** |  | **AA** | **PA** | **BA** | **PA/AA** | **BA/AA** |
|  | **T cells** | n.s. | r = - 0.289 ; p = 0.02 ; n = 88 | n.s. | r = - 0.288 ; p = 0.006 ; n = 88 | n.s. |
|  | **CD4 T cells** | n.s. | r = - 0.289 ; p = 0.004 ; n = 100 | r = - 0.243 ; p = 0.015 ; n = 99 | r = - 0.293 ; p = 0.003 ; n = 100 | n.s. |
|  | **reg CD4 T cells** | n.s. | n.s. | r = - 0.241 ; p = 0.016 ; n = 99 | n.s. | n.s. |
|  | **naive CD4 T cells** | n.s. | r = - 0.352 ; p = 0.0003 ; n = 99 | r = - 0.267 ; p = 0.008 ; n = 98 | r = - 0.358 ; p = 0.0002 ; n = 99 | r = - 0.233 ; p = 0.021 ; n = 98 |
|  | **CM CD4 T cells** | n.s. | n.s. | r = - 0.208 ; p = 0.040 ; n = 98 | r = - 0.243 ; p = 0.015 ; n = 99 | r = - 0.232 ; p = 0.021 ; n = 98 |
|  | **TD CD4 T cells** | n.s. | n.s. | n.s. | n.s. | n.s. |
|  | **EM CD4 T cells** | n.s. | n.s. | n.s. | n.s. | n.s. |
|  |  |  |  |  |  |  |
|  |  |  |  |  |  |  |
| **MS4** |  | **AA** | **PA** | **BA** | **PA/AA** | **BA/AA** |
|  | **T cells** | n.s. | n.s. | n.s. | n.s. | n.s. |
|  | **CD4 T cells** | n.s. | n.s. | n.s. | n.s. | n.s. |
|  | **reg CD4 T cells** | n.s. | r = - 0.494 ; p = 0.008 ; n = 28 | r = - 0.496 ; p = 0.007 ; n = 28 | n.s. | n.s. |
|  | **naive CD4 T cells** | n.s. | n.s. | n.s. | n.s. | n.s. |
|  | **CM CD4 T cells** | n.s. | n.s. | n.s. | n.s. | n.s. |
|  | **TD CD4 T cells** | n.s. | n.s. | r = - 0.552 ; p = 0.003 ; n = 26 | n.s. | n.s. |
|  | **EM CD4 T cells** | n.s. | n.s. | n.s. | n.s. | n.s. |
|  |  |  |  |  |  |  |
| AA: acetate; PA: propionate; BA: butyrate; Reg: regulators; CM: central memory; TD: terminally diferentiated; EM: effector memory. | | | | | |  |
| Correlations were assessed by using the Spearman's rank correlation coefficient (r); n.s.: not significant. | | | | |  |  |
| Colored cells indicate Spearman's rank correlation coefficient >0.3 or <-0.3 | | | |  |  |  |

1. *CD8 T lymphocytes*

| **HC** |  | **AA** | **PA** | **BA** | **PA/AA** | **BA/AA** |
| --- | --- | --- | --- | --- | --- | --- |
|  | **CD8 T cells** | n.s. | n.s. | n.s. | n.s. | n.s. |
|  | **naive CD8 T cells** | r = 0.378 ; p = 0.004 ; n = 84 | r = -0.249 ; p = 0.02 ; n = 84 | n.s. | r = -0.440 ; p = 2*10-5 ; n = 84 | r = -0.293 ; p = 0.008 ; n = 79 |
|  | **CM CD8 T cells** | n.s. | r = -0.290 ; p = 0.007 ; n = 84 | n.s. | r = -0.295 ; p = 0.007 ; n = 84 | n.s. |
|  | **TD CD8 T cells** | n.s. | n.s. | n.s. | n.s. | n.s. |
|  | **EM CD8 T cells** | n.s. | n.s. | n.s. | n.s. | n.s. |
|  |  |  |  |  |  |  |
|  |  |  |  |  |  |  |
| **MS** |  | **AA** | **PA** | **BA** | **PA/AA** | **BA/AA** |
|  | **CD8 T cells** | n.s. | r = - 0.204 ; p = 0.02 ; n = 124 | n.s. | r = - 0.250 ; p = 0.005 ; n = 124 | n.s. |
|  | **naive CD8 T cells** | n.s. | n.s. | n.s. | n.s. | n.s. |
|  | **CM CD8 T cells** | n.s. | n.s. | n.s. | n.s. | n.s. |
|  | **TD CD8 T cells** | n.s. | n.s. | n.s. | n.s. | n.s. |
|  | **EM CD8 T cells** | n.s. | n.s. | n.s. | n.s. | n.s. |
|  |  |  |  |  |  |  |
|  |  |  |  |  |  |  |
| **MS2** |  | **AA** | **PA** | **BA** | **PA/AA** | **BA/AA** |
|  | **CD8 T cells** | n.s. | n.s. | n.s. | n.s. | n.s. |
|  | **naive CD8 T cells** | n.s. | r = -0.204 ; p = 0.044 ; n = 98 | n.s. | r = -0.255 ; p = 0.026 ; n = 98 | n.s. |
|  | **CM CD8 T cells** | n.s. | n.s. | n.s. | n.s. | n.s. |
|  | **TD CD8 T cells** | n.s. | n.s. | n.s. | n.s. | n.s. |
|  | **EM CD8 T cells** | n.s. | n.s. | n.s. | n.s. | n.s. |
|  |  |  |  |  |  |  |
|  |  |  |  |  |  |  |
| **MS4** |  | **AA** | **PA** | **BA** | **PA/AA** | **BA/AA** |
|  | **CD8 T cells** | n.s. | n.s. | n.s. | n.s. | n.s. |
|  | **naive CD8 T cells** | n.s. | n.s. | n.s. | n.s. | n.s. |
|  | **CM CD8 T cells** | n.s. | n.s. | n.s. | n.s. | n.s. |
|  | **TD CD8 T cells** | n.s. | n.s. | n.s. | n.s. | n.s. |
|  | **EM CD8 T cells** | n.s. | n.s. | n.s. | n.s. | n.s. |
|  |  |  |  |  |  |  |
| AA: acetate; PA: propionate; BA: butyrate; Reg: regulators; CM: central memory; TD: terminally diferentiated; EM: effector memory. | | | | | |  |
| Correlations were assessed by using the Spearman's rank correlation coefficient (r); n.s.: not significant. | | | | |  |  |
| Colored cells indicate Spearman's rank correlation coefficient >0.3 or <-0.3 | | | |  |  |  |
|  |  |  |  |  |  |  |

1. *B lymphocytes*

| **HC** |  | **AA** | **PA** | **BA** | **PA/AA** | **BA/AA** |
| --- | --- | --- | --- | --- | --- | --- |
|  | **B cells** | n.s. | r = 0.336 ; p = 0.003 ; n = 76 | n.s. | r = 0.318 ; p = 0.005 ; n = 76 | r = 0.260 ; p = 0.03 ; n = 72 |
|  | **Trans B cells** | n.s. | n.s. | n.s. | n.s. | r = -0.270 ; p = 0.02 ; n = 72 |
|  | **naive B cells** | n.s. | r = 0.376 ; p = 0.0009 ; n = 75 | r = 0.279 ; p = 0.02 ; n = 71 | r = 0.353 ; p = 0.002 ; n = 75 | r = 0.310 ; p = 0.009 ; n = 71 |
|  | **mem B cells** | n.s. | n.s. | n.s. | n.s. | n.s. |
|  | **Plasmablasts** | n.s. | r = - 0.303 ; p = 0.008 ; n = 76 | n.s. | r = - 0.232 ; p = 0.04 ; n = 76 | n.s. |
|  |  |  |  |  |  |  |
|  |  |  |  |  |  |  |
| **MS** |  | **AA** | **PA** | **BA** | **PA/AA** | **BA/AA** |
|  | **B cells** | n.s. | r = 0.283 ; p = 0.001 ; n = 133 | r = 0.227 ; p = 0.001 ; n = 132 | r = 0.245 ; p = 0.004 ; n = 133 | n.s. |
|  | **Trans B cells** | n.s. | r = 0.179 ; p = 0.03 ; n = 125 | n.s. | n.s. | n.s. |
|  | **naive B cells** | n.s. | r = 0.316 ; p = 0.001 ; n = 106 | r = 0.251 ; p = 0.01 ; n = 105 | r = 0.240 ; p = 0.01 ; n = 110 | n.s. |
|  | **mem B cells** | n.s. | r = 0.258 ; p = 0.004 ; n = 125 | r = 0.186 ; p = 0.03 ; n = 124 | r = 0.272 ; p = 0.002 ; n = 124 | r = 0.200 ; p = 0.04 ; n = 124 |
|  | **Plasmablasts** | r = 0.377 ; p = 1*10-5 ; n = 127 | n.s. | n.s. | r = - 0.194 ; p = 0.03 ; n = 131 | r = - 0.302 ; p = 0.001 ; n = 126 |
|  |  |  |  |  |  |  |
|  |  |  |  |  |  |  |
| **MS2** |  | **AA** | **PA** | **BA** | **PA/AA** | **BA/AA** |
|  | **B cells** | n.s. | r = 0.280 ; p = 0.005 ; n = 100 | r = 0.210 ; p = 0.037 ; n = 99 | r = 0.233 p = 0.019 | n.s. |
|  | **Trans B cells** | n.s. | n.s. | r = 0.220 ; p = 0.029 ; n = 98 | n.s. | n.s. |
|  | **naive B cells** | n.s. | r = 0.255 ; p = 0.016 ; n = 88 | n.s. | r = 0.222 ; p = 0.038 ; n = 88 | n.s. |
|  | **mem B cells** | n.s. | r = 0.237 ; p = 0.018 ; n = 99 | n.s. | r = 0.243 ; p = 0.016 ; n = 99 | n.s. |
|  | **Plasmablasts** | r = 0.283 ; p = 0.004 ; n = 100 | n.s. | n.s. | n.s. | n.s. |
|  |  |  |  |  |  |  |
|  |  |  |  |  |  |  |
| **MS4** |  | **AA** | **PA** | **BA** | **PA/AA** | **BA/AA** |
|  | **B cells** | n.s. | n.s. | n.s. | n.s. | n.s. |
|  | **Trans B cells** | n.s. | n.s. | n.s. | n.s. | n.s. |
|  | **naive B cells** | n.s. | r = 0.599 ; p = 0.009 ; n = 18 | r = 0.490 ; p = 0.039 ; n = 18 | n.s. | n.s. |
|  | **mem B cells** | n.s. | n.s. | n.s. | n.s. | n.s. |
|  | **Plasmablasts** | r = 0.606 ; p = 0.001 ; n = 27 | n.s. | n.s. | r = - 0.502 ; p = 0.008 ; n = 27 | r = - 0.666 ; p = 0.0001 ; n = 27 |
|  |  |  |  |  |  |  |
| AA: acetate; PA: propionate; BA: butyrate; Reg: regulators; Trans: transitionals; Mem: memory. | | | |  |  |  |
| Correlations were assessed by using the Spearman's rank correlation coefficient (r); n.s.: not significant. | | | | |  |  |
| Colored cells indicate Spearman's rank correlation coefficient >0.3 or <-0.3 | | | |  |  |  |

1. *NK Cells*

| **HC** |  | **AA** | **PA** | **BA** | **PA/AA** | **BA/AA** |
| --- | --- | --- | --- | --- | --- | --- |
|  | **NK cells** | n.s. | r = 0.360 ; p = 0.002 ; n = 75 | n.s. | r = 0.378 ; p = 0.0008 ; n = 75 | n.s. |
|  | **CD56^dim^ NK cells** | n.s. | r = 0.284 ; p = 0.01 ; n = 76 | n.s. | r = 0.290 ; p = 0.01 ; n = 76 | n.s. |
|  | **CD56^bright^ NK cells** | n.s. | n.s. | n.s. | n.s. | n.s. |
|  | **NKT cells** | n.s. | r = 0.233 ; p = 0.04 ; n = 76 | n.s. | r = 0.263 ; p = 0.02 ; n = 76 | n.s. |
|  |  |  |  |  |  |  |
|  |  |  |  |  |  |  |
| **MS** |  | **AA** | **PA** | **BA** | **PA/AA** | **BA/AA** |
|  | **NK cells** | n.s. | n.s. | n.s. | n.s. | n.s. |
|  | **CD56^dim^ NK cells** | r = - 0.268 ; p = 0.002 ; n = 128 | r = 0.216 ; p = 0.01 ; n = 128 | r = 0.233 ; p = 0.009 ; n = 127 | r = 0.437 ; p = 3*10-7 ; n = 128 | r = 0.416 ; p = 1*10-6 ; n = 127 |
|  | **CD56^bright^ NK cells** | r = 0.443 ; p = 4*10-7 ; n = 121 | n.s. | n.s. | r = - 0.261 ; p = 0.004 ; n = 121 | r = - 0.338 ; p = 0.0002 ; n = 120 |
|  | **NKT cells** | n.s. | n.s. | n.s. | n.s. | n.s. |
|  |  |  |  |  |  |  |
|  |  |  |  |  |  |  |
| **MS2** |  | **AA** | **PA** | **BA** | **PA/AA** | **BA/AA** |
|  | **NK cells** | n.s. | n.s. | n.s. | n.s. | n.s. |
|  | **CD56^dim^ NK cells** | r = -0.211 ; p = 0.036 ; n = 99 | r = 0.282 ; p = 0.005 ; n = 99 | r = 0.246 ; p = 0.014 ; n = 98 | r = 0.416 ; p = 0.0001 ; n = 99 | r = 0.359 ; p = 0.0002 ; n = 98 |
|  | **CD56^bright^ NK cells** | r = 0.389 ; p = 0.00008 ; n = 97 | n.s. | n.s. | r = -0.239 ; p = 0.018 ; n = 97 | r = - 0.323 ; p = 0.001 ; n = 96 |
|  | **NKT cells** | n.s. | n.s. | n.s. | n.s. | n.s. |
|  |  |  |  |  |  |  |
|  |  |  |  |  |  |  |
| **MS4** |  | **AA** | **PA** | **BA** | **PA/AA** | **BA/AA** |
|  | **NK cells** | n.s. | n.s. | n.s. | n.s. | n.s. |
|  | **CD56^dim^ NK cells** | r = - 0.411 ; p = 0.017 ; n = 29 | n.s. | n.s. | n.s. | r = 0.534 ; p = 0.003 ; n = 29 |
|  | **CD56^bright^ NK cells** | r = 0.541 ; p = 0.006 ; n = 24 | n.s. | n.s. | n.s. | n.s. |
|  | **NKT cells** | n.s. | n.s. | n.s. | n.s. | n.s. |
|  |  |  |  |  |  |  |
| AA: acetate; PA: propionate; BA: butyrate; NK: natural killer cells; NKT: natural killer T cells. | | | |  |  |  |
| Correlations were assessed by using the Spearman's rank correlation coefficient (r); n.s.: not significant. | | | | |  |  |
| Colored cells indicate Spearman's rank correlation coefficient >0.3 or <-0.3 | | | |  |  |  |

1. *Monocytes*

| **HC** |  | **AA** | **PA** | **BA** | **PA/AA** | **BA/AA** |
| --- | --- | --- | --- | --- | --- | --- |
|  | **Monocytes** | n.s. | n.s. | r = - 0.357 ; p = 0.001 ; n = 79 | n.s. | r = - 0.264 ; p = 0.02 ; n = 79 |
|  | **Classical Monocytes** | n.s. | n.s. | r = - 0.368 ; p = 0.002 ; n = 71 | n.s. | r = - 0.286 ; p = 0.02 ; n = 71 |
|  | **Intermediate Monocytes** | n.s. | n.s. | n.s. | n.s. | n.s. |
|  | **Non classical Monocytes** | n.s. | n.s. | n.s. | n.s. | n.s. |
|  |  |  |  |  |  |  |
|  |  |  |  |  |  |  |
| **MS** |  | **AA** | **PA** | **BA** | **PA/AA** | **BA/AA** |
|  | **Monocytes** | n.s. | n.s. | r = 0.173 ; p = 0.05 ; n = 129 | n.s. | n.s. |
|  | **Classical Monocytes** | n.s. | n.s. | r = 0.195 ; p = 0.046 ; n = 105 | n.s. | n.s. |
|  | **Intermediate Monocytes** | n.s. | n.s. | n.s. | n.s. | n.s. |
|  | **Non classical Monocytes** | n.s. | n.s. | r = 0.231 ; p = 0.018 ; n = 105 | n.s. | n.s. |
|  |  |  |  |  |  |  |
|  |  |  |  |  |  |  |
| **MS2** |  | **AA** | **PA** | **BA** | **PA/AA** | **BA/AA** |
|  | **Monocytes** | n.s. | n.s. | n.s. | n.s. | n.s. |
|  | **Classical Monocytes** | n.s. | n.s. | n.s. | n.s. | n.s. |
|  | **Intermediate Monocytes** | n.s. | n.s. | n.s. | n.s. | n.s. |
|  | **Non classical Monocytes** | n.s. | n.s. | n.s. | n.s. | n.s. |
|  |  |  |  |  |  |  |
|  |  |  |  |  |  |  |
| **MS4** |  | **AA** | **PA** | **BA** | **PA/AA** | **BA/AA** |
|  | **Monocytes** | n.s. | n.s. | n.s. | n.s. | n.s. |
|  | **Classical Monocytes** | n.s. | n.s. | n.s. | n.s. | n.s. |
|  | **Intermediate Monocytes** | n.s. | r = 0.545 ; p = 0.019 ; n = 18 | n.s. | r = 0.523 ; p = 0.026 ; n = 18 | n.s. |
|  | **Non classical Monocytes** | n.s. | n.s. | n.s. | n.s. | n.s. |
|  |  |  |  |  |  |  |
| AA: acetate; PA: propionate; BA: butyrate. | |  |  |  |  |  |
| Correlations were assessed by using the Spearman's rank correlation coefficient (r); n.s.: not significant. | | | | |  |  |
| Colored cells indicate Spearman's rank correlation coefficient >0.3 or <-0.3 | | | |  |  |  |

1. *Dendritic cells*

| **HC** |  | **AA** | **PA** | **BA** | **PA/AA** | **BA/AA** |
| --- | --- | --- | --- | --- | --- | --- |
|  | **Dendritic cells (D.C.)** | r = - 0.250 ; p = 0.03 ; n = 75 | r = 0.398 ; p = 0.0004 ; n = 75 | n.s. | r = 0.451 ; p = 4*10-5 ; n = 75 | n.s. |
|  | **Plasmacytoid D.C.** | n.s. | n.s. | n.s. | n.s. | n.s. |
|  | **Myeloid D.C.** | n.s. | r = 0.327 ; p = 0.004 ; n = 75 | n.s. | r = 0.326 ; p = 0.004 ; n = 75 | n.s. |
|  |  |  |  |  |  |  |
|  |  |  |  |  |  |  |
| **MS** |  | **AA** | **PA** | **BA** | **PA/AA** | **BA/AA** |
|  | **Dendritic cells (D.C.)** | n.s. | r = 0.335 ; p = 0.0004 ; n = 106 | r = 0.277 ; p = 0.004 ; n = 105 | n.s. | n.s. |
|  | **Plasmacytoid D.C.** | r = 0.234 ; p = 0.01 ; n = 106 | r = 0.247 ; p = 0.01 ; n = 106 | n.s. | n.s. | n.s. |
|  | **Myeloid D.C.** | n.s. | r = 0.253 ; p = 0.009 ; n = 106 | r = 0.244 ; p = 0.01 ; n = 105 | n.s. | n.s. |
|  |  |  |  |  |  |  |
|  |  |  |  |  |  |  |
| **MS2** |  | **AA** | **PA** | **BA** | **PA/AA** | **BA/AA** |
|  | **Dendritic cells (D.C.)** | n.s. | r = 0.342 ; p = 0.001 ; n = 88 | r = 0.277 ; p = 0.009 ; n = 87 | r = 0.263 ; p = 0.013 ; n = 88 | n.s. |
|  | **Plasmacytoid D.C.** | n.s. | r = 0.250 ; p = 0.019 ; n = 88 | n.s. | n.s. | n.s. |
|  | **Myeloid D.C.** | n.s. | r = 0.241 ; p = 0.0023 ; n = 88 | r = 0.278 ; p = 0.009 ; n = 87 | r = 0.259 ; p = 0.015 ; n = 88 | n.s. |
|  |  |  |  |  |  |  |
|  |  |  |  |  |  |  |
| **MS4** |  | **AA** | **PA** | **BA** | **PA/AA** | **BA/AA** |
|  | **Dendritic cells (D.C.)** | r = 0.529 ; p = 0.024 ; n = 18 | n.s. | n.s. | n.s. | n.s. |
|  | **Plasmacytoid D.C.** | n.s. | n.s. | n.s. | n.s. | n.s. |
|  | **Myeloid D.C.** | r = 0.589 ; p = 0.01 ; n = 18 | n.s. | n.s. | n.s. | n.s. |
|  |  |  |  |  |  |  |
| AA: acetate; PA: propionate; BA: butyrate. | | |  |  |  |  |
| Correlations were assessed by using the Spearman's rank correlation coefficient (r); n.s.: not significant. | | | | |  |  |
| Colored cells indicate Spearman's rank correlation coefficient >0.3 or <-0.3 | | | |  |  |  |
