# Supplementary material for: Short‐chain fatty acids in multiple sclerosis: Associated with disability, number of T2 lesions, and inflammatory profile
Source: Ann Clin Transl Neurol. 2025 Mar 3;12(3):478–90. doi: 10.1002/acn3.52259 (PMC11920722; doi:10.1002/acn3.52259)
Supplement: Supplementary file 5 — Table S4. Correlations for the SCFA and their ratios with the expression of intracellular cytokines in MS patients and controls. [file ACN3-12-478-s002.docx]

**Supplementary Table 4.** Correlations for the SCFA and their ratios with the expression of intracellular cytokines in MS patients and controls.

1. *CD4 T lymphocytes*

| **HC** |  | **AA** | **PA** | **BA** | **PA/AA** | **BA/AA** |
| --- | --- | --- | --- | --- | --- | --- |
|  | **CD4 IFN-γ** | n.s. | r = 0.248 ; p = 0.04 ; n = 69 | n.s. | n.s. | n.s. |
|  | **CD4 GM-CSF** | n.s. | r = - 0.514 ; p = 6*10-6 ; n = 69 | n.s. | r = - 0.330 ; p = 0.006 ; n = 69 | n.s. |
|  | **CD4 TNF-α** | n.s. | n.s. | n.s. | n.s. | n.s. |
|  | **CD4 IL-17** | n.s. | n.s. | n.s. | n.s. | n.s. |
|  |  |  |  |  |  |  |
|  |  |  |  |  |  |  |
| **MS** |  | **AA** | **PA** | **BA** | **PA/AA** | **BA/AA** |
|  | **CD4 IFN-γ** | n.s. | r = - 0.313 ; p = 0.003 ; n = 91 | r = - 0.253 ; p = 0.016 ; n = 90 | r = - 0.333 ; p = 0.0012 ; n = 91 | r = - 0.244 ; p =0.02 ; n = 90 |
|  | **CD4 GM-CSF** | n.s. | r = - 0.444 ; p = 2*10-5 ; n = 85 | r = - 0.378 ; p = 3*10-4 ; n = 84 | r = - 0.473 ; p = 5*10-6 ; n = 85 | r = - 0.426 ; p =5*10-5 ; n = 84 |
|  | **CD4 TNF-α** | n.s. | r = - 0.305 ; p = 0.003 ; n = 91 | r = - 0.293 ; p = 0.005 ; n = 90 | r = - 0.383 ; p = 0.0002 ; n = 91 | r = - 0.324 ; p = 0.002 ; n = 90 |
|  | **CD4 IL-17** | n.s. | n.s. | n.s. | n.s. | n.s. |
|  |  |  |  |  |  |  |
|  |  |  |  |  |  |  |
| **MS2** |  | **AA** | **PA** | **BA** | **PA/AA** | **BA/AA** |
|  | **CD4 IFN-γ** | n.s. | r = - 0.308 ; p = 0.009; n = 70 | n.s. | r = - 0.348 ; p = 0.003 ; n = 70 | n.s. |
|  | **CD4 GM-CSF** | r = 0.252 ; p = 0.04 ; n = 67 | r = - 0.441 ; p = 0.0001 ; n = 67 | r = - 0.343 ; p = 0.005 ; n = 66 | r = - 0.504 ; p = 0.0001 ; n = 67 | r = - 0.432 ; p = 0.0002 ; n = 66 |
|  | **CD4 TNF-α** | n.s. | r = - 0.320 ; p = 0.007 ; n = 70 | r = - 0.269 ; p = 0.03 ; n = 69 | r = - 0.439 ; p = 0.0001 ; n = 70 | r = - 0.348 ; p = 0.003 ; n = 69 |
|  | **CD4 IL-17** | n.s. | n.s. | r = - 0.260 ; p = 0.03 ; n = 69 | n.s. | n.s. |
|  |  |  |  |  |  |  |
|  |  |  |  |  |  |  |
| **MS4** |  | **AA** | **PA** | **BA** | **PA/AA** | **BA/AA** |
|  | **CD4 IFN-γ** | n.s. | n.s. | n.s. | n.s. | n.s. |
|  | **CD4 GM-CSF** | n.s. | n.s. | n.s. | n.s. | n.s. |
|  | **CD4 TNF-α** | n.s. | n.s. | n.s. | n.s. | n.s. |
|  | **CD4 IL-17** | n.s. | n.s. | n.s. | n.s. | n.s. |
|  |  |  |  |  |  |  |
| AA: acetate; PA: propionate; BA: butyrate; IFN γ: interferon gamma; GM-CSF: granulocyte-macrophage colony-stimulating factor; TNF α: tumor necrosis factor alpha; IL-17: interleukin 17. | | | | | | |
| Correlations were assessed by using the Spearman's rank correlation coefficient (r); n.s.: not significant. | | | | |  |  |
| Colored cells indicate Spearman's rank correlation coefficient >0.3 or <-0.3 | | | |  |  |  |

1. *CD8 T lymphocytes*

| **HC** |  | **AA** | **PA** | **BA** | **PA/AA** | **BA/AA** |
| --- | --- | --- | --- | --- | --- | --- |
|  | **CD8 IFN-γ** | n.s | r = - 0.242 ; p = 0.04 ; n = 69 | r = - 0.351 ; p = 0.004 ; n = 69 | n.s | n.s |
|  | **CD8 GM-CSF** | n.s | n.s | n.s | n.s | n.s |
|  | **CD8 TNF-α** | n.s | n.s | n.s | n.s | n.s |
|  | **CD8 IL-17** | n.s | n.s | n.s | n.s | n.s |
|  |  |  |  |  |  |  |
|  |  |  |  |  |  |  |
| **MS** |  | **AA** | **PA** | **BA** | **PA/AA** | **BA/AA** |
|  | **CD8 IFN-γ** | n.s | r = - 0.314 ; p = 0.003 ; n = 90 | r = - 0.327 ; p = 0.002 ; n = 91 | r = - 0.354 ; p = 0.0009 ; n = 92 | r = - 0.315 ; p = 0.002 ; n = 91 |
|  | **CD8 GM-CSF** | n.s | r = - 0.391 ; p = 0.0002 ; n = 85 | r = - 0.457 ; p = 1*10-5 ; n = 84 | r = - 0.416 ; p = 7*10-5 ; n = 85 | r = - 0.453 ; p = 1*10-5 ; n = 84 |
|  | **CD8 TNF-α** | n.s | r = - 0.276 ; p = 0.009 ; n = 90 | r = - 0.262 ; p = 0.013 ; n = 89 | r = - 0.358 ; p = 0.0005 ; n = 90 | r = - 0.309 ; p = 0.003 ; n = 89 |
|  | **CD8 IL-17** | n.s | n.s | n.s | n.s | n.s |
|  |  |  |  |  |  |  |
|  |  |  |  |  |  |  |
| **MS2** |  | **AA** | **PA** | **BA** | **PA/AA** | **BA/AA** |
|  | **CD8 IFN-γ** | n.s | r = - 0.328 ; p = 0.006 ; n = 70 | r = - 0.291 ; p = 0.015 ; n = 69 | r = - 0.375 ; p = 0.001 ; n = 70 | r = - 0.296 ; p = 0.01 ; n = 69 |
|  | **CD8 GM-CSF** | n.s | r = - 0.424 ; p = 0.0003 ; n = 67 | r = - 0.494 ; p = 0.00002 ; n = 66 | r = - 0.419 ; p = 0.0004 ; n = 67 | r = - 0.426 ; p = 0.0003 ; n = 66 |
|  | **CD8 TNF-α** | n.s | r = - 0.291 ; p = 0.01 ; n = 70 | r = - 0.263 ; p = 0.03 ; n = 69 | r = - 0.425 ; p = 0.0002 ; n = 70 | r = - 0.362 ; p = 0.002 ; n = 69 |
|  | **CD8 IL-17** | n.s | n.s | n.s | n.s | n.s |
|  |  |  |  |  |  |  |
|  |  |  |  |  |  |  |
| **MS4** |  | **AA** | **PA** | **BA** | **PA/AA** | **BA/AA** |
|  | **CD8 IFN-γ** | n.s | n.s | n.s | n.s | n.s |
|  | **CD8 GM-CSF** | n.s | n.s | n.s | n.s | r = - 0.496 ; p = 0.036 ; n = 18 |
|  | **CD8 TNF-α** | n.s | n.s | n.s | n.s | n.s |
|  | **CD8 IL-17** | n.s | n.s | n.s | n.s | n.s |
|  |  |  |  |  |  |  |
| AA: acetate; PA: propionate; BA: butyrate; IFN γ: interferon gamma; GM-CSF: granulocyte-macrophage colony-stimulating factor; TNF α: tumor necrosis factor alpha; IL-17: interleukin 17. | | | | | | |
| Correlations were assessed by using the Spearman's rank correlation coefficient (r); n.s.: not significant. | | | | |  |  |
| Colored cells indicate Spearman's rank correlation coefficient >0.3 or <-0.3 | | | |  |  |  |

1. *B lymphocytes*

| **HC** |  | **AA** | **PA** | **BA** | **PA/AA** | **BA/AA** |
| --- | --- | --- | --- | --- | --- | --- |
|  | **CD19 GM-CSF** | r = - 0.263 ; p = 0.03 ; n = 69 | n.s. | n.s. | n.s. | r = 0.311 ; p = 0.01 ; n = 64 |
|  | **CD19 TNF-α** | n.s. | n.s. | n.s. | n.s. | n.s. |
|  |  |  |  |  |  |  |
|  |  |  |  |  |  |  |
| **MS** |  | **AA** | **PA** | **BA** | **PA/AA** | **BA/AA** |
|  | **CD19 GM-CSF** | r = 0.357 ; p = 0.0006 ; n = 84 | n.s. | n.s. | r = - 0.251 ; p = 0.022 ; n = 84 | r = - 0.278 ; p = 0.011 ; n = 83 |
|  | **CD19 TNF-α** | n.s. | r = - 0.246 ; p = 0.02 ; n = 89 | n.s. | r = - 0.309 ; p = 0.003 ; n = 89 | r = - 0.226 ; p = 0.035 ; n = 88 |
|  |  |  |  |  |  |  |
|  |  |  |  |  |  |  |
| **MS2** |  | **AA** | **PA** | **BA** | **PA/AA** | **BA/AA** |
|  | **CD19 GM-CSF** | r = 0.255 ; p = 0.039 ; n = 66 | n.s. | n.s. | n.s. | n.s. |
|  | **CD19 TNF-α** | n.s. | n.s. | n.s. | r = - 0.312 ; p = 0.009 ; n = 69 | n.s. |
|  |  |  |  |  |  |  |
|  |  |  |  |  |  |  |
| **MS4** |  | **AA** | **PA** | **BA** | **PA/AA** | **BA/AA** |
|  | **CD19 GM-CSF** | r = 0.639 ; p = 0.004 ; n = 18 | n.s. | n.s. | r = - 0.598 ; p = 0.009 ; n = 18 | r = - 0.583 ; p = 0.01 ; n = 18 |
|  | **CD19 TNF-α** | n.s. | n.s. | n.s. | n.s. | n.s. |
|  |  |  |  |  |  |  |
| AA: acetate; PA: propionate; BA: butyrate; IFN γ: interferon gamma; GM-CSF: granulocyte-macrophage colony-stimulating factor; TNF α: tumor necrosis factor alpha. | | | | | | |
| Correlations were assessed by using the Spearman's rank correlation coefficient (r); n.s.: not significant. | | | | |  |  |
| Colored cells indicate Spearman's rank correlation coefficient >0.3 or <-0.3 | | | |  |  |  |
